# Phospholipid-driven conformational switching of HCV NS5A links protein folding to replication membrane remodeling

**DOI:** 10.1126/sciadv.aeb8863

**Published:** 2026-04-03

**Authors:** Anna V. Bulankina, Rebecca M. Richter, James H. Nettles, Daisuke Yamane, Christian Grimm, Yasaman Karami, Richard A. Stanton, Bianca Introini, Jonas Hermann, Hanaa Charif, Mia S. König, Claudia Stroß, Cristina Ortiz, Nico Kraus, Daniel Wood, Facundo Galceran, Rupert Abele, Bernard Maigret, Raymond F. Schinazi, Stefan Zeuzem, Ricardo M. Biondi, MinKyung Yi, Robert Tampé, Mikhail Kudryashev, Christoph Welsch

**Affiliations:** ^1^ Goethe University Frankfurt, University Hospital, Medical Clinic 1, Frankfurt/Main, Germany.; ^2^ Center for ViroScience and Cure, Laboratory of Biochemical Pharmacology, Department of Pediatrics, Emory University School of Medicine and Children’s Healthcare of Atlanta, Atlanta, GA, USA.; ^3^ Nettles Consulting LLC, Savannah, GA, USA.; ^4^ Tokyo Metropolitan Institute of Medical Science, Tokyo, Japan.; ^5^ Lorraine University, LORIA Institute, CNRS, INRIA, Nancy, France.; ^6^ Max Planck Institute of Biophysics, Frankfurt/Main, Germany.; ^7^ Buchmann Institute of Molecular Life Sciences, Frankfurt/Main, Germany.; ^8^ Instituto de Fisiología, Biología Molecular y Neurociencias (IFIBYNE), Buenos Aires, Argentina.; ^9^ Institute of Biochemistry, Biocenter, Goethe University, Frankfurt/Main, Germany.; ^10^ Facultad de Ciencias Exactas y Naturales, Universidad de Buenos Aires, Buenos Aires, Argentina.; ^11^ Department of Microbiology and Immunology, University of Texas Medical Branch at Galveston, Galveston, TX, USA.; ^12^ Max Delbrück Center for Molecular Medicine (MDC), Berlin, Germany.; ^13^ Institute of Medical Physics and Biophysics, Charité-Universitätsmedizin Berlin, Berlin, Germany.; ^14^ Goethe University Frankfurt, University Hospital, Molecular Hepatology and Inflammation Research, Frankfurt/Main, Germany.

## Abstract

Phospholipids are essential for RNA virus replication, yet their role in modulating conformational dynamics of membrane-associated viral proteins remains poorly understood. For NS5A, a key replication factor of hepatitis C virus, previous crystallographic models fail to capture the lipid-driven conformational mechanics we uncover here. Using structural informatics and biochemical probing of pharmacophore-guided mutants in defined lipid environments, we evaluated competing NS5A domain 1 dimerization models. Our data reveal an alternative membrane-specific fold stabilized by polyproline hinges and phospholipids (PIPs) such as phosphatidylinositol-4-phosphate, a host lipid enriched at replication membranes. PIP binding promotes a conformational switch that drives dimerization, linking lipid sensing to membrane remodeling and host factor recruitment. This reciprocal mechanism—where a lipid allosterically modulates a viral protein that reshapes membranes—is blocked by the antiviral pibrentasvir. These findings define a lipid-driven structural switch that governs NS5A pleiotropy and highlight dynamic lipid-protein interfaces as targets for antiviral intervention.

## Teaser

A host lipid flips a viral protein’s shape to control membrane remodeling during virus replication.

## INTRODUCTION

Phospholipids are key regulators of RNA virus replication, facilitating protein-membrane interactions, stabilizing membrane curvature, and supporting the assembly of replication complexes (RCs) (*1*). In hepatitis C virus (HCV) infection, the association between the host lipid phosphatidylinositol-4-phosphate (PI4P) and the viral nonstructural protein 5A (NS5A) is critical for RC formation (*2*–*5*). NS5A recruits the lipid kinase PI4KIIIα to elevate PI4P levels, driving the formation of double-membrane vesicles (DMVs), the hallmark of the HCV replication platform (*2*–*6*). While NS5A is the target of highly potent antivirals (*7*), the structural mechanisms underlying its membrane interaction, inhibitor sensitivity, and functional versatility remain poorly defined (*8*–*11*).

**Fig. 1.**
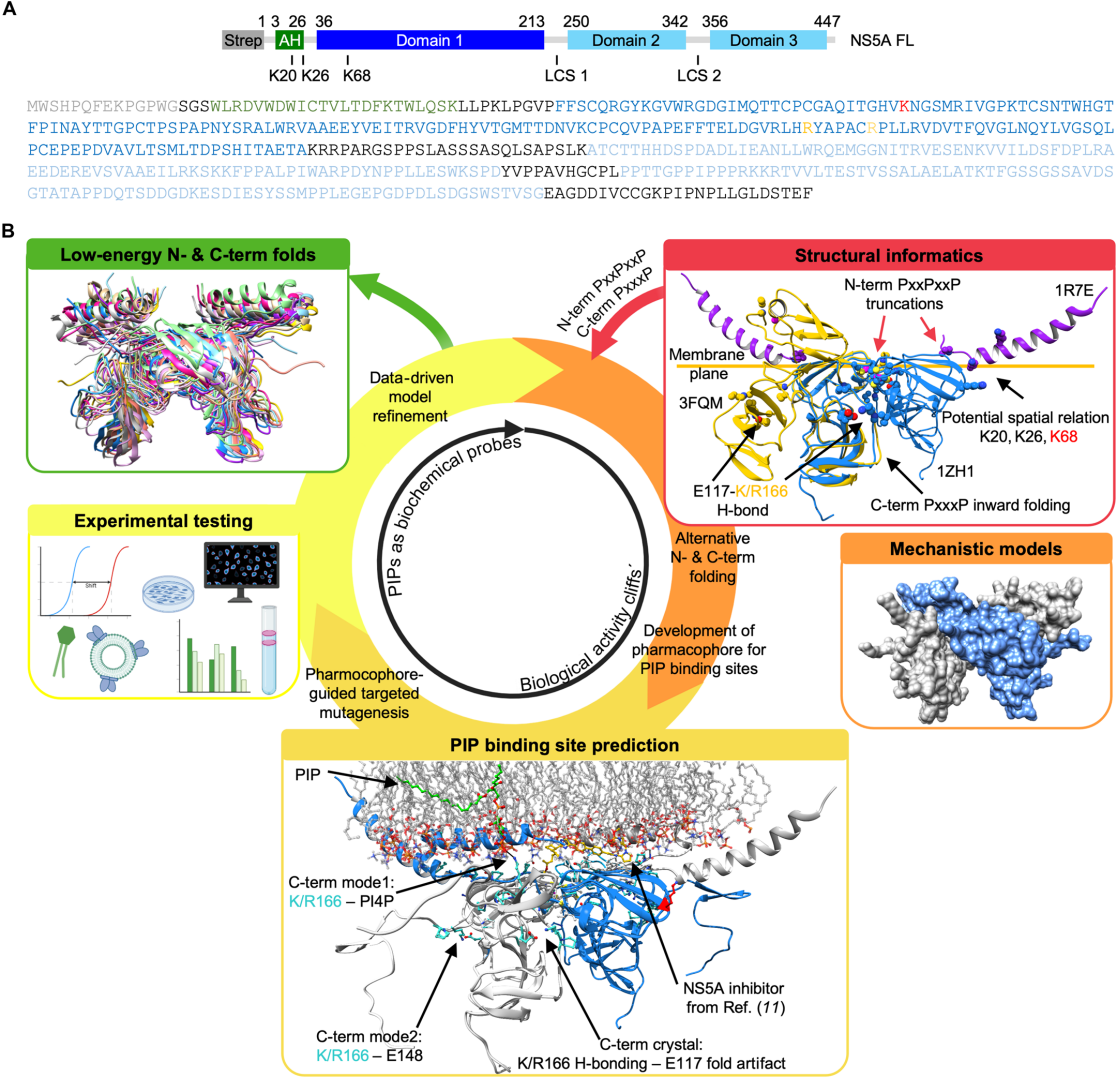
Workflow for modeling and testing dynamic amphiphilic protein environments. (**A**) Domain architecture and amino acid sequence of NS5A (from N- to C-term): Strep affinity-tag (Strep, gray), AH (green), linker region (residues 27 to 35, not indicated), D1 (blue), D2, and D3 (light blue), the latter separated by low complexity sequences (LCS) 1 and 2. (**B**) Structural informatics workflow to evaluate competing models of NS5A-D1 dimerization and folding, using phospholipids as biochemical probes. This framework integrates lipid-mediated probing with computational modeling to assess biology-driven molecular rearrangements and the associated protein conformational space. Red and orange box: Membrane-aligned overlay of experimental structural fragments from nuclear magnetic resonance and x-ray studies (*8*, *9*, *12*). Torsional flexibility within NS5A-D1 (residues 1 to 170) was examined using MODELLER (*16*), systematically sampling rotations within the polyproline linker (residues 160 to 170) to generate low-energy conformations linking the Zn^2+^-binding core to the terminal regions. The orientation of the AH follows the membrane-bound geometry reported by Nettles *et al.* (*11*) and Penin *et al.* (*12*). Light orange box: Identification of a PIP-binding pharmacophore based on the spatial clustering of residues capable of electrostatic interactions with negatively charged phospholipid headgroups. Yellow box: Structural models were used as hypothesis-generating tools to guide mutagenesis and interpret biochemical data. Pharmacophore residues were validated by site-directed mutagenesis across multiple protein constructs and lipid environments. Green box: Model refinement informed by experimental PIP-binding assays and protein-unfolding data. Created in BioRender. Welsch, C. (2025) https://BioRender.com/b54u968.

**Fig. 2.**
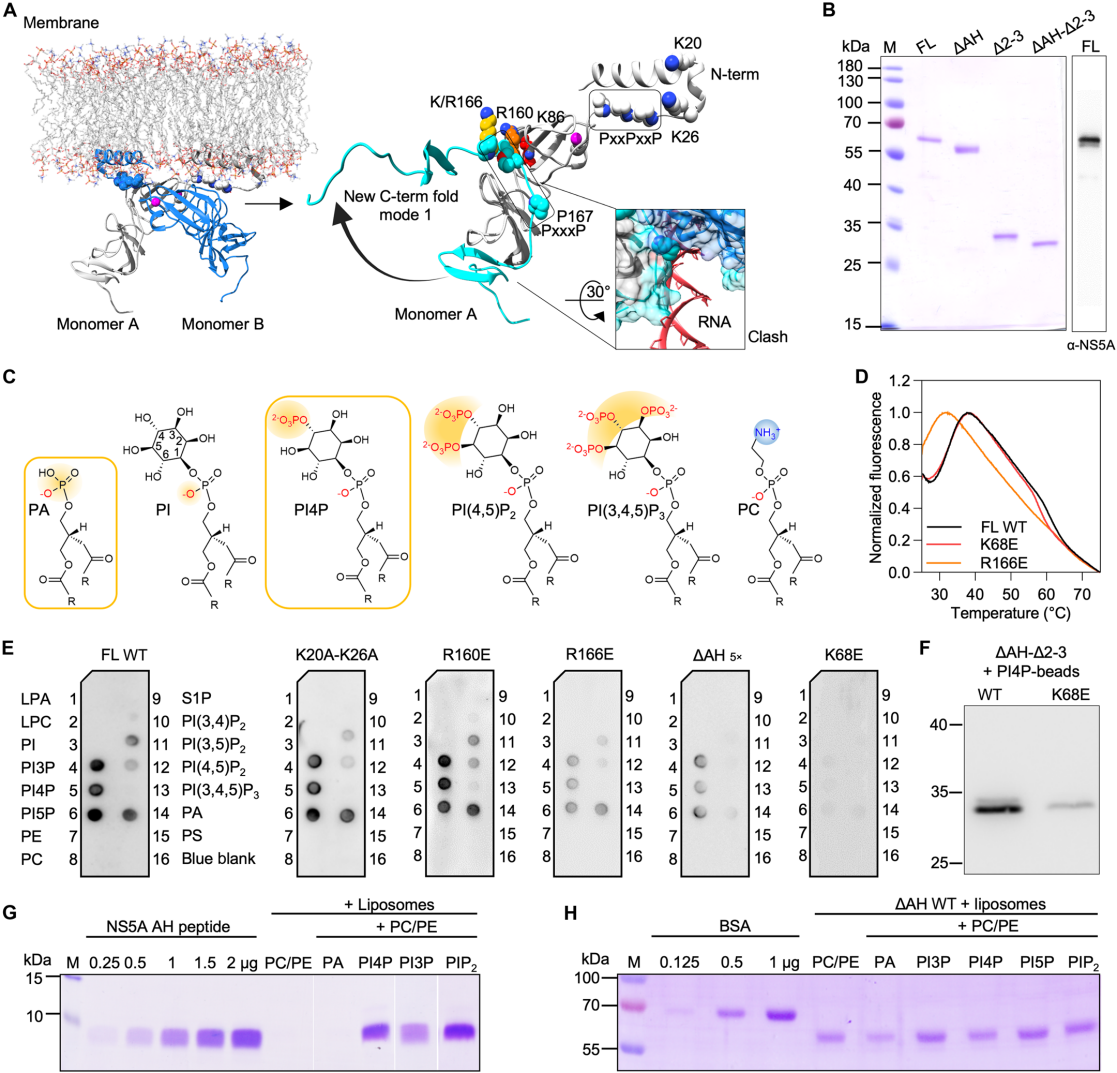
Structural informatics approach and selective phospholipid binding. (**A**) Theoretical AH-D1 model from Nettles *et al.* (*11*) attached to a membrane; D1 dimer from PDB 1ZH1 (*9*) (gray and blue) and AHs from PDB 1R7E and 1R7F (*12*) (left) with putative steric clashes of RNA modeled into the binding groove (inset). Zn^2+^ ions are shown in magenta and proline hinge region in Corey-Pauling-Koltun (CPK) and new C-term fold model (cyan) based on PDB 1ZH1. Residues selected for mutagenesis are shown in CPK (right). (**B**) SDS-PAGE of purified FL WT NS5A, truncation constructs, and Western blotting with anti-NS5A antibody. (**C**) Selected lipid molecules (from PLOA, Fig. 2E) and charge distribution required for binding to NS5A. Lipids with most efficient binding are highlighted by orange boxes; negatively charged phosphate groups (yellow), positively charged headgroups (blue). (**D**) TSA of FL WT NS5A, K68E, and R166E mutant protein. (**E**) Protein-lipid overlay assays (PLOAs) with phospholipid-binding patterns with fivefold molar amount needed for ΔAH. (**F**) Pull-down assay of ΔAH-Δ2-3 WT and K68E mutant with PI4P beads. (**G**) NS5A-AH standard and peptide recruited to and extracted from liposomes with a different lipid composition. (**H**) Recruitment of ΔAH to and extracted from liposomes of the indicated lipid composition. *n* = 3 biologically independent samples (strips/wells/gels) for (D) to (H).

NS5A is a multidomain protein composed of an N-terminal amphipathic helix (AH) (*12*), a structured domain 1 (D1), and two intrinsically disordered regions (D2 and D3) (Figs. 1A and 2A and figs. S1 and S2) (*13*, *14*). Crystal structures of D1 have revealed diverse dimeric arrangements but were based on truncated constructs lacking the membrane context (*8*, *9*, *15*). As a result, the role of phospholipids in modulating NS5A structure and function remains unclear. In this study, we address this gap by modeling and experimentally validating phospholipid-induced conformational changes in full-length NS5A and truncated constructs in defined membrane environments (Fig. 1B and fig. S1).

We set out to define how phospholipids regulate the conformational and functional dynamics of the NS5A protein during RC formation in HCV infected cells. Previous crystal structures suggested an internal hydrogen bond linking residues 117 and 166 (Fig. 1B bottom), folding the C terminus of D1 into a putative RNA binding groove (Fig. 2A, inset) (*8*, *9*, *15*). However, these structures were derived from truncated constructs and likely miss membrane-driven conformational states. In this study, we focused on dissecting the biological mechanisms governing NS5A-phospholipid interactions. The structural models were used to inform and test specific mechanistic hypotheses on how phospholipid binding shapes NS5A conformation at the membrane. We hypothesized that the D1 C terminus can adopt an alternative orientation, rotating toward the membrane in response to lipid binding. Building on integrative structural informatics models of the N-terminal AH combined with D1 in a dimeric model (fig. S1) (*11*, *12*), we predicted an alternative D1 fold, stabilized by a conserved polyproline (PxxxP) motif and spanning both D1 termini and the Zn^2+^-binding core (Figs. 1B and 2A and figs. S1 and S2). This model suggested a phospholipid-binding pharmacophore, which we validated through mutagenesis and lipid-binding assays (Fig. 2). Our results show that phosphoinositide (PIP) binding drives conformational switching in NS5A, linking protein folding to membrane curvature and revealing a lipid-dependent mechanism for organizing viral RC architecture.

## RESULTS

### NS5A directly engages phospholipids via its AH and D1

To dissect lipid-binding properties of NS5A, we generated and purified a panel of recombinant HCV genotype 1b NS5A constructs, including full-length wild type (FL WT), as well as truncations lacking the AH (ΔAH), domains 2 and 3 (Δ2-3), or both (ΔAH-Δ2-3) (Fig. 2B and fig. S3). NS5A-AH was synthesized as a labeled peptide. We first used a protein-lipid overlay assay (PLOA), which detects high-affinity binding to immobilized lipids (Fig. 2, C and E, and fig. S4, A and B). Full-length NS5A showed selective binding to phosphatidylinositol monophosphates (PI3P, PI4P, and PI5P) and phosphatidic acid (PA) and weaker affinity for bisphosphates (PIP_2_) but not the triphosphorylated PIP (Fig. 2, C and E). To validate these interactions in a membrane context and further investigate weaker phospholipid binders, we reconstituted NS5A constructs into phospholipid liposomes and assessed membrane association via flotation assays (Fig. 2, G and H, and fig. S4). We found that both the AH peptide and D1 bound strongly to phosphatidylinositol monophosphates such as PI4P, as well as to PI(4,5)P_2_-containing liposomes, whereas binding to PA was not detected with AH alone (Fig. 2, G and H). The deletion of D2 and D3 (Δ2-3) retained the selectivity profile in PLOA (fig. S4B) but showed decreased binding, indicating a role in providing affinity to phospholipid binding but not in the selectivity toward their headgroup. Furthermore, we confirmed that the AH participates in the interaction with PA since the removal of the AH from the NS5A construct reduced binding to PA (fig. S4B). The remaining binding of monophosphorylated PIPs to this construct further confirmed the important participation of the D1 domain in the interaction with PI3P, PI4P, and PI5P. Consistent results were obtained using PI4P-containing liposomes for NS5A recruitment and glycerol gradient fractionation [fig. S4, D (left) and F]. Together, these findings show that NS5A engages phospholipids through distinct structural elements. For the physiologically highly relevant PI4P, recognition appears to involve two components: (i) nonspecific membrane affinity provided by D2 and D3 and (ii) enhanced headgroup-specific affinity conferred by the AH and D1.

### An alternative folding model positions phospholipid-binding residues at the membrane interface

Crystallographic models of NS5A D1 lack membrane context (*8*, *9*, *15*). On the basis of a previously published AH-D1 model (*11*), we hypothesized that the D1 C-terminal region may adopt alternative conformations in vivo that could facilitate phospholipid binding during RC formation (Figs. 1B and 2A). We used MODELLER (*16*) to systematically explore feasible torsional rotations within the polyproline linker (residues 160 to 170) and identify low-energy conformations consistent with our biochemical observations. In existing structures, an internal hydrogen bond between E117 and K166 tethers the D1 C terminus toward the RNA binding groove, implying a possible steric clash with the RNA [Figs. 1B (bottom) and 2A (inset)] (*8*, *9*, *15*). Releasing this interaction allows K166 and the D1 C-terminal tail to reorient toward the membrane, potentially contributing to the formation of an unrecognized phospholipid-binding surface (Fig. 2A and movie S1). Supporting this reorientation, we identified a conserved PxxxP motif (residues 160 to 170; fig. S2B) that can function as a flexible hinge, enabling rotational positioning of the D1 C-terminal tail parallel to the membrane (Fig. 2A). This configuration aligns positively charged residues—R160 and K/R166 of the D1 C terminus, and K68 of the Zn^2+^-binding core—within interaction distance of phospholipid headgroups, in register with the N-terminal PxxPxxP motif and the K20-K26 pincer in the AH (Fig. 2A and fig. S2) (*17*). Using a stepwise modeling approach of lowest energy folds (Fig. 1B), we defined a composite phospholipid-binding pharmacophore and tested its relevance through targeted mutagenesis and biochemical probing with defined lipid species.

### A composite interface centered on K68 mediates phospholipid binding

To functionally test the predicted pharmacophore, we assessed the effects of targeted mutations on NS5A folding and phospholipid binding. Thermal shift assays (TSAs) confirmed that the D1 residue K68 critically supports phospholipid binding to NS5A: Its substitution (K68E; charge reversing) preserved the protein overall fold but nearly abolished phospholipid binding in overlay and liposome-based assays (Fig. 2, D to F, and fig. S4D). In contrast, R166E decreased protein thermal stability, consistent with its role in conformational switching. Although binding efficiency was reduced, phospholipid specificity remained unaffected. R160E had no effect on phospholipid binding (Fig. 2E). Notably, PI4P-dependent recruitment of the ΔAH and ΔAH-Δ2-3 construct to liposomes was markedly enhanced, while K68E abolished PI4P bead binding [Fig. 2F and fig. S4, C (right) and D (right)]. Mutations of AH pincer residues (K20A and K26A) (*17*) selectively impaired binding to bulky PIP_2_ species but not to monophosphates, confirming differential headgroup sensitivity (Fig. 2, C and E). Full AH deletion abrogated PIP_2_ binding and reduced overall affinity, requiring higher protein input for comparable PLOA signal (Fig. 2E). The primary determinant of higher selectivity to monophosphorylated PIPs resides in the D1 domain. Our data support a distributed phospholipid-binding interface spanning the AH and D1, with K68 acting as the key residue mediating PIP recognition (fig. S8C).

### PIP binding to K68 supports NS5A localization and HCV replication

**Fig. 3.**
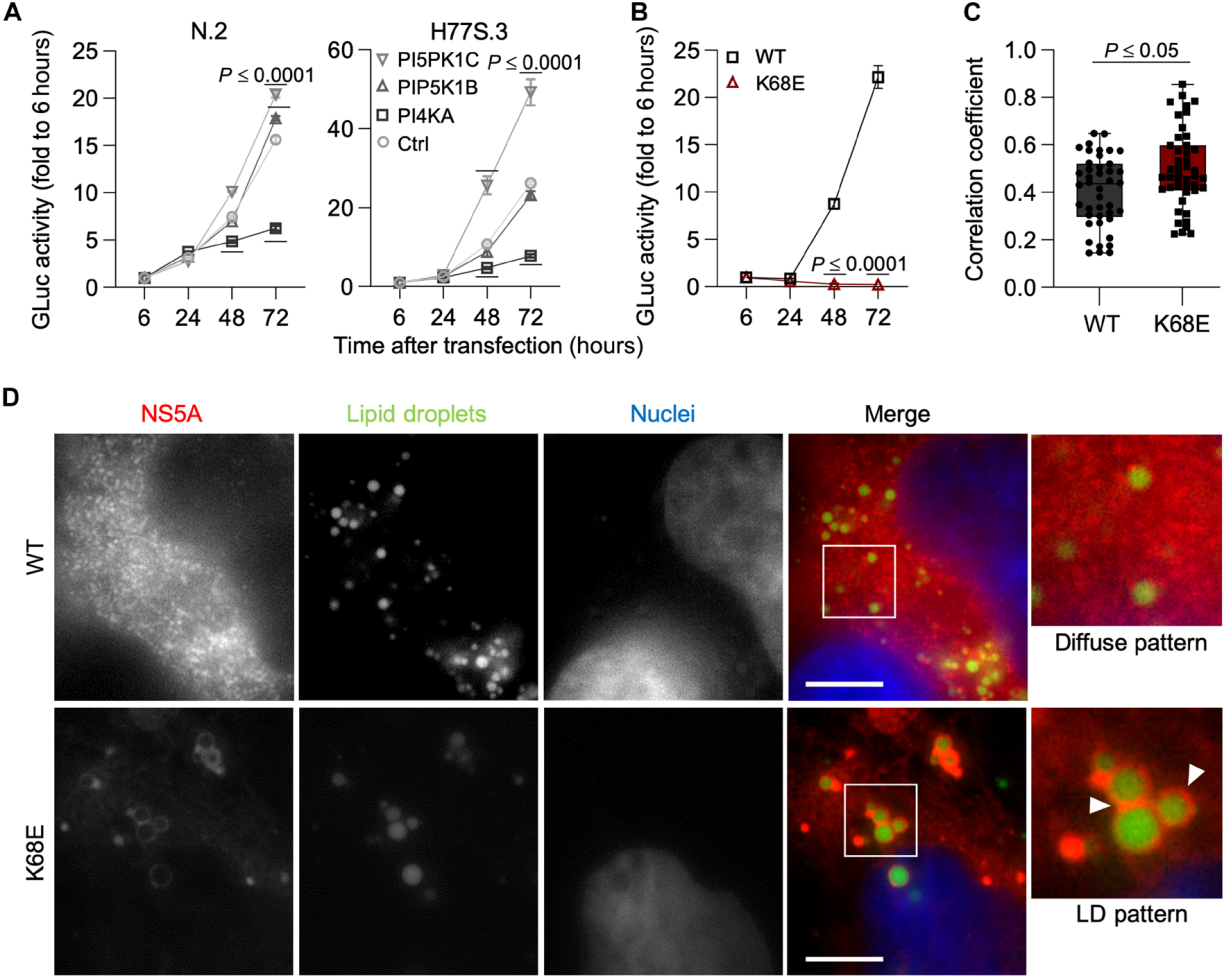
Impact of PI4P metabolism and K68 interaction on viral replication. (**A**) Replication capacity of N.2 (gt1b) and H77S.3 (gt1a), respectively, in cells transfected with indicated siRNAs in Huh7.5 cell culture, as determined by secreted Gaussia luciferase (GLuc) activity at indicated time points. (**B**) Replication capacity of K68E mutant virus in an N.2 genetic background. (**C**) Pearson’s correlation for the association of NS5A with LDs [shown in (D)]. (**D**) Immunofluorescence images showing the subcellular distribution of NS5A 48 hours after transfection of Huh7 cells with WT (diffuse distribution) or K68E (LD pattern); NS5A in red, LDs in green, and cell nuclei in blue. Data shown are mean ± SEM for (A) and (B); quartiles (dashed lines) and median (solid lines) for (C). *n* = 3 or 5 biologically independent samples (wells). Two-way analysis of variance (ANOVA) for (A) and (B) and one-way ANOVA for (C).

During HCV infection, PI4P levels are increased by the recruitment of PI4KIIIα through NS5A (*2*). Therefore, we examined the functional significance of PI4P interaction with NS5A during the viral replication cycle. Small interfering RNA (siRNA)–mediated knockdown of PI4KA, the kinase that generates PI4P, significantly reduced replication of both genotype 1a and 1b HCV strains, highlighting the genotype-independent requirement for PI4P (Fig. 3A and fig. S6, A to C). Conversely, depletion of PIP5K1C, which converts PI4P into PI(4,5)P_2_, increased viral replication due to intracellular PI4P accumulation (Fig. 3A and fig. S6, A to C). These effects were mirrored at the structural level: Both charge-reversing (K68E) and charge-neutralizing (H66Q-K68Q) mutations at K68 abolished replication entirely (Fig. 3B and fig. S6D), consistent with our biochemical and folding data identifying K68 as the key residue in the phospholipid-binding site (Fig. 2, D to F, and fig. S4, C and D).

We further demonstrated that PIP binding at K68 contributes to the subcellular localization of NS5A in transfected cells. While WT NS5A localized diffusely in the cytoplasm, consistent with endoplasmic reticulum (ER)–associated replication sites, K68 mutants relocalized to lipid droplets (LDs), as shown by fluorescence colocalization with LD markers (Fig. 3D). Pearson correlation analysis confirmed a significant shift toward LD association in the mutants (Fig. 3, C and D, and fig. S6, E and F). K68 contributes to NS5A interactions with PIPs, including PI4P, and helps regulate NS5A subcellular localization and HCV replication. These effects in infected cells are more complex and likely arise from multiple, overlapping mechanisms involving ER association and RC assembly, rather than exclusive PI4P specificity. The data support a model in which PIP binding at K68 contributes to anchoring NS5A at RCs, while loss of this interaction facilitates its relocalization toward LDs, consistent with a replication-to-assembly switch in the viral life cycle.

### PIP binding induces conformational transitions in the AH-D1 region

**Fig. 4.**
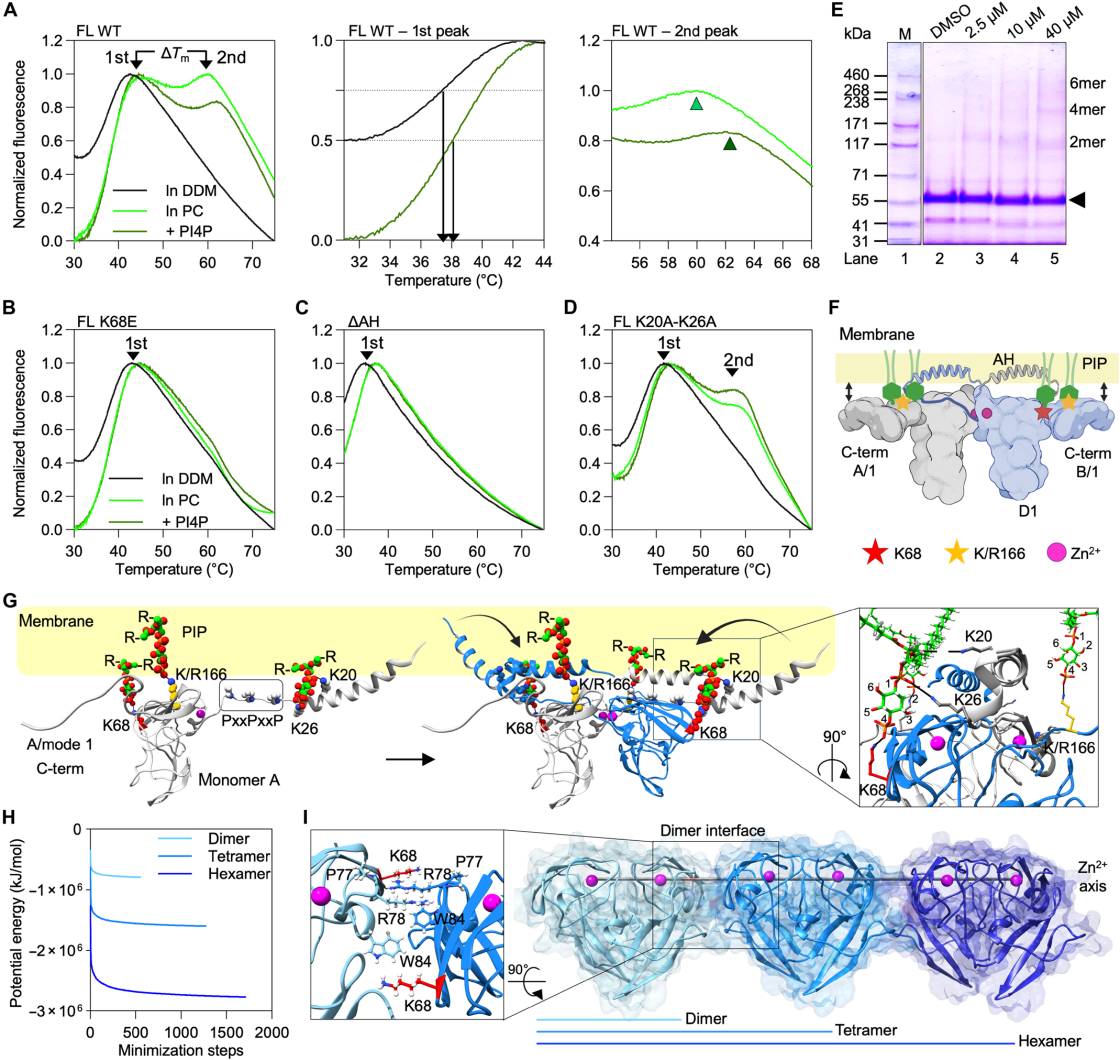
Effect of phospholipids on NS5A fold and AH-D1 conformational rearrangements. (**A** to **D**) Normalized fluorescence emission curves of relipidated NS5A constructs and effect of phospholipids on thermal stability. Melting curves in the presence of detergent or lipids: (A) FL WT (left). Temperature shift with PI4P, first peak (middle) and second peak (right), (B) FL K68E mutant, (C) ΔAH construct, and (D) FL K20A-K26A mutant (**E**). Cross-linking of FL WT with different BMH concentrations. (**F**) Mechanistic model of AH and D1 protein core aligned in a symmetric dimer fold to the membrane surface with PIP headgroups as spacers. Created in BioRender. Welsch, C. (2025) https://BioRender.com/lwebmk7. (**G**) PIP binding and NS5A-AH rearrangement parallel to the membrane. Close-up view (right) of PIP binding sites; PIP-bound K68 of monomer A in lateral packing with the AH pincer of the extended N terminus from monomer B. This inward AH folding stabilizes the overall protein fold, resulting in a lower-energy state (Fig. 4H). PI4P was used as model ligand. (**H**) Steepest-descent energy calculation for NS5A dimer, tetramer, and hexamer model. (**I**) NS5A oligomer model and Zn^2+^-ion axis. Close-up view (left) showing residue alignment in the dimer interface. In (A) to (E), *n* = 3 biologically independent samples (wells/gels).

To explore how specific phospholipid interactions affect NS5A stability and folding, we reconstituted purified FL-NS5A and selected mutants into mixed micelles with phosphatidylcholine (PC) or the biologically relevant PI4P (fig. S5C) and performed TSA. In detergent [n-dodecyl-β-D-maltopyranoside (DDM)], FL NS5A showed a single unfolding transition (*T*_m_ = 36.3° ± 1.4°C), consistent with a monomeric, detergent-solubilized conformation (Fig. 4A, left). Size-exclusion chromatography coupled to multi-angle light scattering (SEC-MALS) confirmed that FL NS5A in DDM exists as a monomer bound to detergent micelles (fig. S4G). This is also consistent with analytical ultracentrifugation data by Kwon *et al.* (*18*), supporting the interpretation of the TSA data.

Relipidation with PC or PC/PI4P induced a second, higher-temperature melting peak (*T*_m2_ ≈ 54° to 60°C), indicative of a more stable protein state (*19*, *20*), that we interpreted as oligomerization. In support of our interpretation, Jirasko *et al.* (*21*) previously demonstrated NS5A self-association in a lipid environment. Above we showed that PC bound to NS5A with low affinity, as we did not observe binding by PLOA but readily observed binding to NS5A in liposome interaction assays (Fig. 2, E and H). Here, we observed that PC slightly shifted the monomeric peak (*T*_m1_ ≈ 38°C) (Fig. 4A, left and middle). Notably, in the presence of PC with the addition of PI4P, the oligomeric peak was further shifted to higher *T*_m_ (Fig. 4A, left and right). Together, on the basis of our data and on previous knowledge, we interpret the first peak as the NS5A monomer and the second peak as the more stable oligomer, while we interpret the small shifts as the stabilization of the monomer or oligomer by the interaction with PC or PC plus PI4P. Notably, we show that the inclusion of PI4P did not modify the stability of the first peak but specifically increased the temperature stability of the oligomeric form of NS5A, Δ*T*_m2_ by ~1.5°C, suggesting that PI4P binds to and stabilizes the higher-order conformation of NS5A (Fig. 4A, right). In contrast, the K68E mutation abolished the second peak entirely (Fig. 4B) but conserved the ability to shift the thermal stability of the monomeric form, implicating that the mutation affected NS5A oligomerization but did not affect the binding to PC. This result enabled us to establish that K68 facilitates a conformational rearrangement of NS5A that underlies its dimerization/oligomerization, independent of its involvement in lipid binding.

As expected from our previous experiments showing that the mutation disrupts the binding to monophosphorylated PIPs, PI4P did not thermally stabilize K68E. Similarly, ΔAH mutants failed to form the higher-*T*_m_ species (Fig. 4C), while the AH pincer mutant K20A-K26A (*17*) only eliminated the PI4P-specific *T*_m2_ shift but not oligomer formation per se (Fig. 4D).

To link these observations to the lipid-binding assays, it is important to note that liposome recruitment and PLOA measure the membrane affinity of NS5A, whereas TSA reports how these membrane interactions influence NS5A’s conformational ensemble. Lipids that promote membrane engagement also stabilize distinct structural states of NS5A, which appear in TSA as shifts in the monomeric (*T*_m1_) and oligomeric (*T*_m2_) melting transitions. PI4P-containing compositions, which strongly recruit NS5A in vitro, selectively stabilize the oligomeric state, while the K68E and ΔAH mutants lose this specific stabilization. Other PIPs may also contribute to stabilizing oligomeric states, indicating that PI4P is modulatory but not unique. Thus, the TSA data, PLOA, and liposome recruitment assays (LRAs) converge mechanistically: Together, they reveal how specific phospholipids and residues shape NS5A’s membrane association and conformational switching.

Cross-linking confirmed the simultaneous presence of NS5A monomers, dimers, and higher-order species (Fig. 4E), consistent with phospholipid-stabilized oligomerization. The ~22°C difference between the two melting peaks likely reflects an equilibrium between these structural states, with the higher-temperature form being considerably more stable. Beldar *et al.* showed similar equilibrium between NS5A monomers, transient dimers, and higher-order multimers (*22*).

Our results show that relipidation stabilizes the NS5A fold through at least three interdependent mechanisms: (i) shifts in the conformational ensemble, (ii) promotion of dimer and oligomer formation, and (iii) PIP-induced structural rearrangements. Together, these data indicate that K68 and the AH act cooperatively to promote the conformational transition required for high-affinity PIP binding.

### A structural switch links PIP binding to NS5A multimerization

**Fig. 5.**
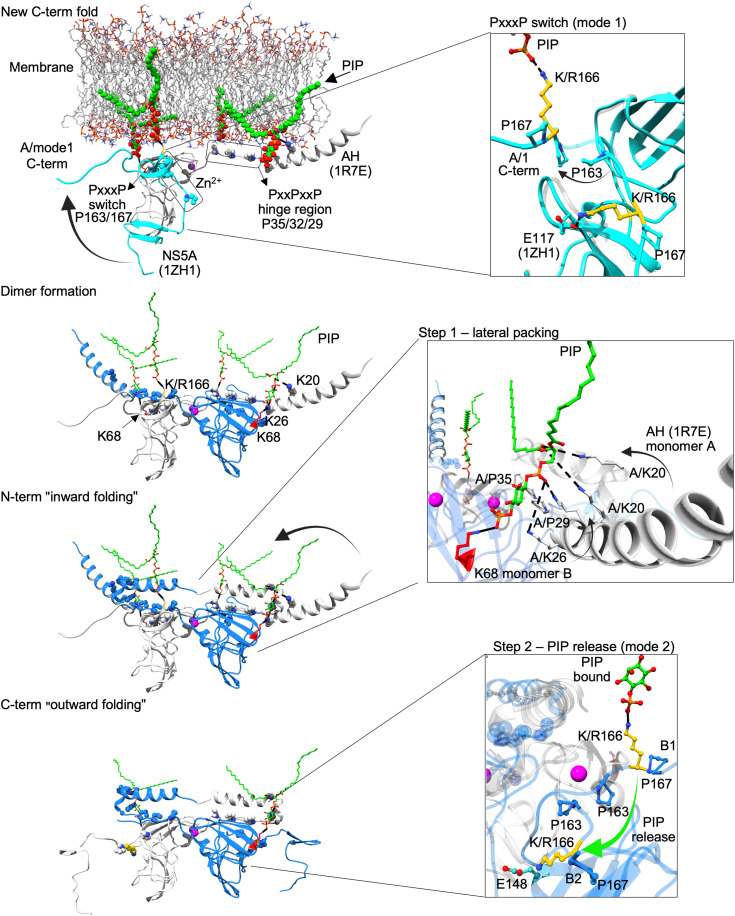
Step-wise mechanistic model of NS5A dimer formation and PIP-regulated AH-D1 folding. This model illustrates a plausible mechanistic sequence in which PIP binding induces conformational changes in NS5A that promote dimer formation and higher-order multimerization relevant for RNA binding. The process is governed by a dynamic “folding switch” involving the AH, the D1 domain, and the PxxxP hinge motif (residues 163 to 167). PIP interacts with positively charged residues in the AH (K20 and K26) and the D1 core (K68), stabilizing membrane anchoring. A second PIP-binding site involves residue K/R166, whose side chain aligns perpendicularly with the PIP headgroup to position the D1 C terminus at the membrane (mode 1). This orientation is enabled by the PxxxP hinge (see the top inset). Next, PIP binding at K/R166 stabilizes a planar geometry of the D1 domain, aligning its N- and C-terminal regions and priming the protein for symmetric dimer assembly. Upon dimerization, the N-terminal AHs fold inward toward the dimer core (step 1; middle inset), forming a compact membrane-proximal interface. In the subsequent conformational transition (step 2; bottom inset), PIP is released from K/R166. This enables the D1 C terminus to rotate outward (mode 2), driven by the flexibility of the PxxxP hinge. K/R166 forms a stabilizing intramolecular salt bridge with E148 (see the bottom inset), locking the extended dimer interface. This conformational switch corresponds to the E117-E148 interaction observed in PDB 1ZH1 (Fig. 1B) (*9*), known to stabilize NS5A dimers and essential for HCV replication (*47*). PI4P was used as model ligand to illustrate the mechanisms.

Our structural informatics model reveals a unique interlocked NS5A dimer geometry, in which the two monomers adopt a perpendicular/parallel arrangement (Fig. 4F). This configuration enables the N-terminal AHs of NS5A to fold inward via a conserved PxxPxxP motif, while the D1 C termini extend outward through the flexible PxxxP hinge (Fig. 4G and fig. S7). Conformational transitions likely occur in two distinct steps, driven by PIP binding at residues K68 and K/R166 (Figs. 2A, 4G, and 5 and fig. S7). To mechanistically dissect these NS5A folding transitions, we combined TSAs, lipid-binding experiments, and structure-based modeling. By using interacting phospholipids and biologically defined “activity cliffs” (*11*) as functional probes, we were able to map the conformational rearrangements relevant to NS5A’s membrane-associated activity (Fig. 1B).

A key finding integrated into the structural informatics model was the strong interaction between monophosphorylated PIPs and residues K68 and K/R166 (Fig. 2, E and F), which directly influenced the folding of both the AH and the C-terminal region of D1 (Figs. 2D and 4, A, B, and G, and fig. S7). Conversely, mutations of K68 or R166 impaired PIP binding and destabilized fold formation (Fig. 2, D to F). Our model predicts that phospholipid-binding residues—K68 in the D1 core and K/R166 in the D1 C terminus, together with K20 and K26 in the AH (*17*)—are spatially aligned within the membrane plane. These residues are coordinated via interactions with the PIP headgroups, acting as molecular spacers that stabilize the AH-D1 membrane interface and promote conformational interlocking between NS5A monomers (Fig. 4, F and G, and fig. S7). The central structural element enabling the dynamic of NS5A is the polyproline hinge (PxxxP, residues 160 to 170), which links the Zn^2+^-binding core to the flexible C-terminal tail of D1 (Fig. 4G and fig. S7). This hinge permits rotational movement, allowing residue K/R166 to adopt two conformational modes: In mode 1, the residue interacts with PIPs at the membrane interface; in mode 2, it disengages from the membrane and folds inward to form an intramolecular salt bridge with E148, thereby extending the dimer interface (Figs. 4, F and G, and 5).

Steepest descent simulations of the folding transitions revealed distinct energy drops upon formation of the PIP-bound dimer interface, followed by further stabilization during higher-order oligomerization (Fig. 4H). These transitions function mechanistically as a “folding lever”: PIP binding first stabilizes the membrane-associated dimer in a low-energy conformation (Fig. 4, G and H), corresponding to increased thermal stability observed in TSA measurements (Fig. 4A). Dimerization brings PIP-bound K68 of NS5A monomer A into lateral contact with the AH pincer (*17*) of monomer B [Figs. 4G (right) and 5 and fig. S7], promoting inward folding of both AHs toward the dimer core. This lateral packing stabilizes the membrane-proximal AH-D1 interface (step 1: lateral packing) (Fig. 5 and fig. S8A) and defines a low-energy folding state (Fig. 4H). The experimental data support this folding model: The increased thermal stability of this conformation is reflected by the upward shift in *T*_m2_ in TSA (Fig. 4A), indicating the formation of a stabilized fold. Truncation of the AH reduced lipid-binding affinity (Fig. 2E) and abolished the second melting transition (*T*_m2_) in TSA (Fig. 4C), indicative of impaired higher-order folding. The release of PIP from residue R166 enables outward refolding of the D1 C terminus and permits multimer growth (step 2: release) [Fig. 5 (bottom), fig. S9A, and movie S2]. Notably, the two polyproline motifs—P29-P32-P35 in the AH and P163-P167 at the D1 hinge—act as structural fulcrums that facilitate these monomeric folding transitions from mode 1 to mode 2 (Fig. 5). The transitions enable lateral packing between NS5A dimers and promote higher order multimerization (Fig. 4I and fig. S7), aligning with the dynamic remodeling of HCV replication compartments during the viral life cycle. The outward folding of the D1 C terminus upon PIP release (mode 2) correlates with the active replacement of PI4P by cholesterol in DMVs during HCV replication (*23*). Supporting this model, LRA showed that cholesterol-containing liposomes recruit less AH peptide compared to PI4P-enriched membranes (fig. S6G). This suggests that declining PIP/PI4P levels act as molecular switch, triggering NS5A relocalization from replication compartments (DMVs) to LDs (Fig. 3, C and D, and fig. S6, E and F).

The PIP-stabilized dimer interface features W84 as a central hydrophobic contact point, shielded by residues P77 and R78 (Fig. 4I and fig. S8B). Together, these form a “hitching site” that supports RNA binding groove formation, consistent with structural observations by Zhang *et al.* (*24*). Steepest descent minimization of tetrameric and hexameric assemblies revealed distinct energy drops (Fig. 4H), likely driven by complementary electrostatic interactions between charged surface residues (Fig. 4I and fig. S8A). The extended dimers generated through the switch assemble into linear multimeric arrays that align with RNA duplex geometry, supporting their proposed role in RC formation in HCV infected cells (fig. S9).

### The PIP-K68 interaction modulates NS5A-induced membrane curvature

**Fig. 6.**
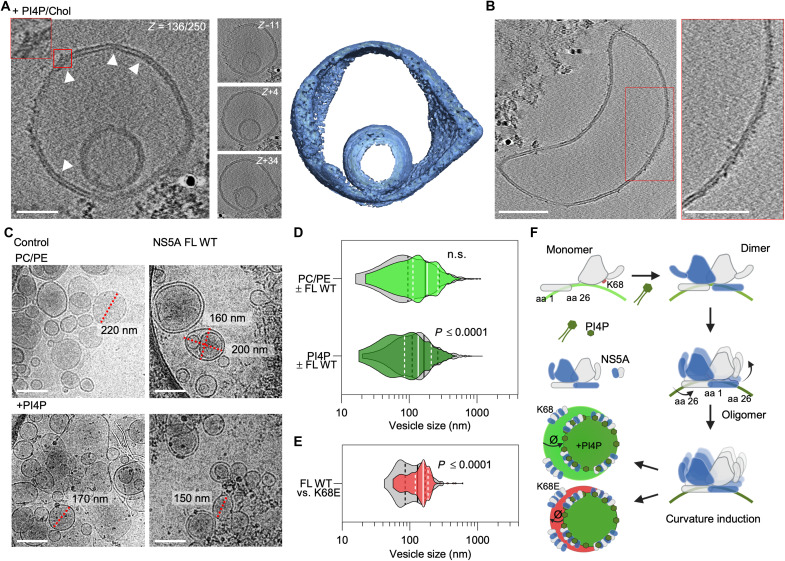
Role of PI4P and K68 in modulating membrane curvature. (**A**) Sections through a cryo–electron tomography (cryo-ET) reconstruction of DMV with reconstituted FL WT protein. Proteinaceous density and sharp edges as part of the membrane remodeling process; red frame inset indicates zoom in. Scale bar, 50 nm. Slice Z − 11 = −1.54 nm, Z + 4 = 0.56 nm, and Z + 34 = +4.76 nm. (**B**) Slice through a cryo-ET reconstruction showing a proteoliposome with longitudinal protein density of reconstituted FL WT NS5A. Scale bars, 100 nm. (**C**) Cryo–electron microscopy (cryo-EM) micrographs of PC/PE and PC/PE/PI4P liposomes in the absence (left) or presence (right) of FL WT NS5A; representative liposomes in red framed insets. Scale bars, 200 nm. (**D**) Violin plots showing the liposome size distribution on a logarithmic scale for FL WT NS5A in PC/PE control (lime green) or PC/PE/PI4P liposomes (green) (*n* = 1076 versus 499 and 1056 versus 580, respectively), control liposomes in gray, or (**E**) FL WT NS5A versus K68E mutant in PI4P-containing liposomes (red) (*n* = 289 versus 161). (**F**) Schematic of AH-D1 lipid interaction and oligomerization with PI4P-dependent curvature induction of WT and K68E. Created in BioRender. Welsch, C. (2025) https://BioRender.com/n96b0vb. Data shown are quartiles (dashed lines) and median (solid lines) for (D) and (E). Mann-Whitney *U* test for (D) and (E). n.s., not significant; aa, amino acid.

Our findings reveal that PIP binding to K68 not only regulates NS5A folding and subcellular localization (Figs. 3, C and D, and 4B) but also directly modulates its capacity to induce membrane curvature. Reconstitution of NS5A into PI4P-containing liposomes (fig. S5B) resulted in the formation of highly curved vesicles, including double- and multimembrane structures (Fig. 6 and fig. S10, A to C), consistent with the ultrastructure of HCV replication organelles (*6*, *25*). PI4P-containing proteoliposomes with FL protein showed lipid bilayers covered with a proteinaceous density, mostly on the outside (Fig. 6, A and B), and “sharp edges” next to this density [Fig. 6A and fig. S10, A (bottom) and D], confirming successful reconstitution. This membrane remodeling activity was strongly diminished in the absence of PI4P or in the K68E mutant, underscoring the requirement for specific phospholipid interactions with NS5A to initiate curvature (Fig. 6, E and F). PI4P enhances the efficiency and curvature of DMV formation, but NS5A alone is sufficient for DMV generation (fig. S10B).

Deletion studies further clarified the structural contributions of NS5A domains: AH facilitates lipid recruitment and vesicle growth (fig. S11), while D2 and D3 promote curvature generation (fig. S10D, E). Only the full-length protein in the presence of PI4P induced the formation of small, uniformly curved vesicles (Fig. 6, C and D), suggesting that PIPs such as PI4P act as a molecular trigger for structural transitions within AH-D1 that drive curvature. In contrast, PI4P-free liposomes or liposomes reconstituted with K68E mutant protein lacked this remodeling capacity (Fig. 6E), highlighting the central role of PIP-K68 binding in enabling NS5A’s membrane-shaping function. These results point to a finely tuned mechanism in which specific phospholipid interactions at the AH-D1 interface control the extent and nature of membrane curvature, supporting a model in which PIP binding coordinates NS5A conformational dynamics with the physical restructuring of host membranes during RC formation (Fig. 6F).

### Pibrentasvir disrupts the PIP-binding interface of NS5A

**Fig. 7.**
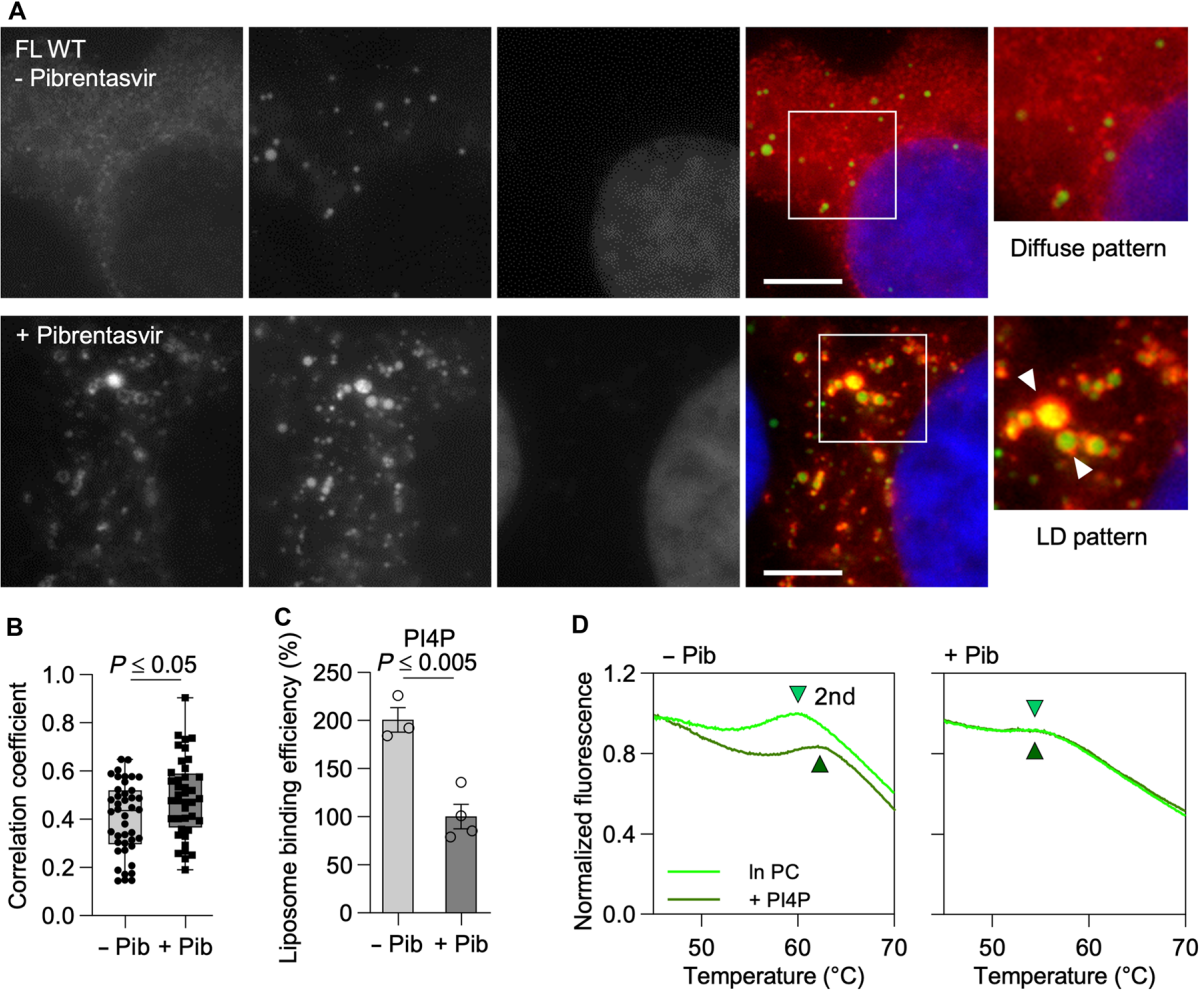
Impact of pibrentasvir on PI4P interaction with AH-D1. (**A**) Immunofluorescence images showing subcellular distribution of NS5A WT 48 hours after transfection of Huh7 cells without pibrentasvir (diffuse distribution, top) or with pibrentasvir (LD pattern, bottom); NS5A (red), LDs (green), and cell nuclei (blue). Scale bar, 5 μm. (**B**) Pearson’s correlation for the association of NS5A with LDs shown in (A). (**C**) LRA of ΔAH WT ± pibrentasvir to PI4P-containing liposomes. (**D**) Normalized fluorescence emission curves of FL WT ± pibrentasvir and effect of the lipid environment on thermal stability (second peak). Data shown are quartiles (dashed lines) and median (solid lines) for (B) and mean ± SEM for (C). *n* = 3 or 5 biologically independent samples (wells/gels). One-way ANOVA for (B) and two-way ANOVA for (C).

To determine whether clinically approved NS5A inhibitors interfere with the phospholipid-binding interface, we assessed the effect of pibrentasvir on NS5A-PIP interactions. Pibrentasvir is a pan-genotypic HCV inhibitor that targets NS5A-D1 (*26*). This class of compounds blocks viral assembly and release and disrupts the formation of intracellular membrane structures required for HCV RNA synthesis (*27*). Treatment of NS5A-transfected cells with pibrentasvir redirected NS5A from ER-derived membranes to LDs (Fig. 7, A and B), a subcellular relocalization associated with the shift from genome replication to virion assembly. Concomitantly, we observed a pronounced reduction in PI4P binding to NS5A in LRAs (Fig. 7C), accompanied by a loss of the PI4P-dependent conformational state required for replication compartment formation. Specifically, pibrentasvir blocked the PI4P-induced thermal shift of the second melting transition (*T*_m2_) in TSA (Fig. 7D), a shift associated with the formation of a PI4P-stabilized higher-order conformation of NS5A. These findings indicate that pharmacological disruption of the PIP-binding interface may underlie the antiviral mechanism of NS5A inhibitors.

## DISCUSSION

Taken collectively, our data establish a direct mechanistic link between host phospholipids such as PI4P and the viral protein NS5A—both key players in the HCV replication cycle. While previous studies described an indirect connection via PI4KIIIα activation (*2*), our findings reveal that NS5A directly senses PIPs, triggering conformational changes that couple protein folding to membrane remodeling and viral RC formation. Existing structural models of NS5A have relied on truncated protein constructs lacking membrane context (*8*, *9*, *15*). Here, we introduce an approach that integrates synthetic biology and structural informatics to model NS5A folding within amphiphilic environments, enabling the prediction of a D1 C-terminal rearrangement and a PIP-binding pharmacophore in D1 and the AH (Fig. 1 and fig. S1). This model builds on the NS5A D1 dimer structure with minimal N-terminal truncation (*9*) and incorporates the inward positioning of the N-terminal AH (*11*), consistent with experimental data, including lipid binding and conformational flexibility, and prior observations on inhibitor sensitivity (*10*). Notably, this configuration supports a potential fold-back mechanism of D2 and D3, as suggested by our structural and biochemical data (Fig. 2, E and H, and fig. S4B). These mechanisms could also facilitate the direct interaction of NS5A with PA, thereby providing a structural basis for its reported role in RC formation, as previously proposed by Tabata *et al.* (*28*). The ability of NS5A to bind other PIPs such as PI3P or PI5P suggests that other phospholipids could also contribute to NS5A membrane association or additional, as-yet undescribed functions. Together, these findings provide a refined mechanistic framework for NS5A function and suggest a membrane-guided refolding mechanism that supports RC formation.

Our findings uncover a PIP-driven structural switch that governs NS5A dimerization, membrane anchoring, and subcellular localization. PIP binding to key residues, K68 at the D1 core and the N-terminal AH pincer (*17*) and K/R166 at the D1 C terminus, stabilizes a paired monomer configuration (Figs. 4F and 5 and fig. S7). This interaction enables a conformational rearrangement that interlocks the N- and C-terminal regions of NS5A, aligning PIP headgroups and protein residues in-plane with the membrane (Fig. 4F) and supporting a dimerization interface that may seed higher-order oligomers (Figs. 4I and 5, and figs. S7 and S9). A similar orientation of the amphipathic helices was observed by Zhang *et al.* (*24*) using an HCV replicase surrogate system. Simultaneously, PI4P binding at the ER induces local membrane bending (Fig. 6, D and E) and anchors NS5A at replication sites. Disrupting this interaction, either through mutagenesis or by using the direct-acting antiviral pibrentasvir, an NS5A inhibitor, causes NS5A to relocalize to LDs (Figs. 3, C and D, and 7, A and B), thereby shifting its role from genome replication to virus assembly. These results establish phospholipids, and among them PI4P, although represented only as small fraction of total phospholipids in eukaryotic cells (*29*, *30*), as a central regulator of NS5A’s conformational state and intracellular trafficking, explaining previously observed localization dynamics and highlighting a lipid-sensing mechanism that couples protein structure to membrane remodeling and functional switching.

In summary, NS5A acts as a dynamic “transformer,” whose structural plasticity and mutual interplay with membrane phospholipids enable context-dependent functions. A conserved phospholipid sensor in the AH-D1 region binds PIPs, autoregulating membrane remodeling and RC formation. Our structural informatics model predicts these folding dynamics, supporting the RNA binding groove hypothesis and the NS5A dimer structure centered on K68-phospholipid interactions (*9*). While more studies are needed, these insights illuminate NS5A inhibitor mechanisms and provide a framework for developing therapies targeting critical lipid interactions.

## MATERIALS AND METHODS

### Experimental design

We designed a biology-guided, integrative structural informatics approach to dissecting the lipid-dependent conformational dynamics of the HCV NS5A D1 in a membrane context (Fig. 1B) using a previously published AH-D1 model reported by Nettles *et al.* (*11*). The overarching goal was to model and experimentally validate mechanistic phospholipid-protein interactions that link NS5A function to host-derived phospholipids critical for viral replication. Central to this approach is a conserved, flexible PxxxP motif (residues 160 to 170; P163xxxP167), which we hypothesized to act as a conformational switch regulating C-terminal folding of D1 upon phospholipid binding.

Using PIPs as biochemical probes of biologically relevant conformational states, we systematically assessed the energetic landscape of D1 folding transitions. Candidate low-energy folds were evaluated for consistency with biochemical data, known drug resistance mutations, and structure-activity relationships (activity cliffs) (*11*). This iterative modeling experimentation framework (Fig. 1B) allowed us to predict phospholipid interaction sites and test their functional relevance using targeted mutagenesis, folding assays, and lipid-binding studies.

### Computational studies

#### Analysis of experimental structures

Structural coordinates of truncated NS5A fragments were downloaded from the RCSB Protein Data Bank (PDB; https://rcsb.org) and used as references for model building, as described by Nettles *et al.* (*11*). Nuclear magnetic resonance structures of gt1a N-terminal AH (residues 1 to 31) were downloaded as PDB 1R7C, 1R7D, 1R7E, 1R7F, and 1R7G (*12*). X-ray crystal structures of D1 from gt1b were downloaded in three dimeric forms, 1ZH1 (residues 36 to 198) (*9*), 3FQM, and 3FQQ (32 to 191) (*8*). Models of D1 with N-terminal inward folding were downloaded from https://pubs.acs.org/doi/full/10.1021/jm501291c (jm501291c_si_003.pdb, jm501291c_si_004.pdb) (*11*). All NS5A structures were aligned using UCSF Chimera version 1.16 for Darwin64 using MatchMaker (*31*). From the structure overlay, the core fold of NS5A-D1 up to residue 160 is consistent in all available experimental models. As was done previously for N-terminal residues 1 to 31 and on the basis of the C-terminal truncated model jm501291c_si_003.pdb (residues 1 to 160) as input template (*11*), we calculated potential alternative folding of C-terminal residues using MODELLER 10.1 (*16*) with default settings (https://salilab.org/modeller/) (Fig. 1B).

#### Development of alternative NS5A fold models

Because the lipid-induced conformational transitions of NS5A involve nonequilibrium processes with substantial torsional motions around proline hinges, conventional molecular dynamics simulations were not used. Instead, torsional flexibility within NS5A D1 (residues 1 to 170) was explored with MODELLER (*16*), which systematically sampled feasible rotations within the polyproline linker (residues 160 to 170) and generated low-energy conformations connecting the Zn^2+^-binding core with the N- and C-terminal regions. These structures were used qualitatively as hypothesis-generating models to guide mutagenesis and to interpret biochemical data. PI4P was included as a model ligand in the figures due to its established relevance to NS5A biology and the improved visibility it offers of residues lining the putative PIP binding pocket. The C-terminal residues from 160 were stepwise extended for each D1 dimer template, 1ZH1, 3FQM, and 3FQQ, and the lowest-energy folds estimated. Two MODELLER folds were considered useful for further modeling/testing based on (i) the ability to form D1 dimers and (ii) their potential to interact with phospholipids. To model these extended C-terminal folds, we used the β sheet folded C-terminal region containing residues 167 to 198 from 1ZH1 aligned to two alternative positions of residue P167 from the selected lowest-energy folds (alternative mode 1 and mode 2 in monomers A and B) (Fig. 5 and fig. S7). Using these fold models, we identified a phospholipid-binding pharmacophore based on the spatial arrangement of residues that enable electrostatic interactions with negatively charged phospholipid headgroups. The pharmacophore residues, 20, 26, 68, 160, and 166, were further tested by site-directed mutagenesis in different protein constructs and lipid contexts. PIP binding data (from PLOA and LRA) for the truncated/mutated NS5A forms, along with protein unfolding data (from TSA), were then used to refine the in silico models (Fig. 1B). The novel N-/C-terminal alignment was then evaluated in a POPC model membrane, popc128a.pdb (*32*). To minimize steric overlaps between the system components, energy minimization was performed for 2000 steps by a steepest descent algorithm. The GROMACS 2021.4 package was used to carry out all simulations (*33*). All structural figures were done with Chimera v1.18 (*31*) and ChimeraX 1.8 (*34*).

### Protein biochemistry

#### Constructs and reagents

His-NS5A-AH peptide with the sequence HHHHHHMSGSWLRDVWDWVCTVLSDFKTWLQSKLLPR was purchased from GenScript. Lipid powder from: 1-palmitoyl-2-oleoyl-sn-glycero-3-phosphocholine (PC), 1-palmitoyl-2-oleoyl-sn-glycero-3-phosphoethanolamine (PE), sheep’s wool cholesterol (Chol), 1,2-dioleoyl-sn-glycero-3-phosphate (sodium salt) (PA), or chloroform/methanol/water (20:9:1) solutions for 1,2-dioleoyl-sn-glycero-3-phospho-(1′-myo-inositol-3′-phosphate) (ammonium salt, PI3P), 1,2-dioleoyl-sn-glycero-3-phospho-(1′-myo-inositol-5′-phosphate) (ammonium salt, PI5P), porcine brain L-α-phosphatidylinositol-4-phosphate (ammonium salt, PI4P), and L-α-phosphatidylinositol-4,5-bisphosphate [ammonium salt, PI(4,5)P_2_] were purchased from Avanti Polar Lipids. Atto550-1,2-Dioleoyl-sn-glycero-3-phosphoethanolamine (Atto550-PE) was purchased from ATTO-TEC.

#### Cloning

FL WT NS5A (amino acids 1 to 447, N.2, gt1b) is cloned between the N-terminal Strep and C-terminal V5 tag into pVL941 baculovirus transfer vector. Truncations, ΔAH (amino acids 31 to 447), Δ2-3 (1 to 249), and ΔAH-Δ2-3 (31 to 249), and point mutations were generated using QuikChange site-directed mutagenesis (Stratagene) according to the manufacturer’s instructions (primers purchased from Eurofins Genomics; table S1). Mutations and truncations were verified by sequencing. For expression in human cells, FL WT and mutants were subcloned into pcDNA 3.1+ via BamHI-HF and XhoI restriction sites after amplification with primers.

#### Sf9 protein production and purification

Transfer vector pVL941-Strep-NS5A-V5 or its variants were used to cotransfect Sf9 insect cells with the baculovirus flashBAC ULTRA genome according to the manufacturer’s instructions (Oxford Expression Technologies). P2 baculovirus was used to infect 900 ml of Sf9 cells at 1.7 × 10^6^ cells/ml in Sf-900 II SFM media (Gibco) with 1% antibiotic-antimycotic solution (Gibco). After 48 hours at 26°C, cells were pelleted and resuspended in hypotonic buffer [10 mM tris-HCl (pH 7.5), 10 mM NaCl, 3 mM MgCl_2_, 2 mM β-mercaptoethanol, phosphatase inhibitor cocktail, and cOmplete EDTA-free Protease Inhibitor Cocktail (Roche)]. All the following steps were performed at 4°C. Cells were sonicated (3×, 10 s), and the postnuclear supernatant was collected after centrifugation for 5 min, 1000*g* and centrifuged again at 145,000*g* for 1 hour. FL, Δ2-3, and ΔAH-Δ2-3 membrane fractions were resuspended in buffer W [100 mM tris-HCl (pH 7.5), 150 mM NaCl, and 2 mM β-mercaptoethanol] using a Dounce homogenizer, snap frozen in liquid nitrogen, and stored at −80°C. ΔAH and ΔAH-Δ2-3 cytosols were snap frozen and stored at −80°C. FL and Δ2-3 membranes were solubilized with 0.5% DDM (Glycon Biochemicals) for 1.5 hours under rotation and centrifuged at 145,000*g* for 1 hour. ΔAH and ΔAH-Δ2-3 cytosol was also centrifuged after thawing. The supernatant was loaded on a Strep-Tactin-Sepharose column (IBA-Lifescience) preequilibrated with buffer E (buffer W supplemented with 5% glycerol and ±0.05% DDM). Column was washed with 15 CV of buffer E (±0.05% DDM), and protein was eluted with buffer E (±0.05% DDM) containing 5 mM desthiobiotin (Sigma-Aldrich). Fractions containing NS5A were pooled and, if necessary, concentrated in Amicon Ultra centrifugation units 2 ml of 10K molecular weight cut-off (MWCO) (Millipore). Proteins were snap frozen in liquid nitrogen and stored at −80°C. Protein was assessed by SDS–polyacrylamide gel electrophoresis (SDS-PAGE) for purity (fig. S2) and concentration using bovine serum albumin (BSA) standards and Fiji/ImageJ (*35*).

#### NS5A inhibitor treatment

A 10 mM stock solution of pibrentasvir (Selleck Chemicals) was prepared in dimethyl sulfoxide (DMSO). During NS5A protein expression, pibrentasvir was added to Sf9 cells at a final concentration of 10 nM together with P2 virus and kept constant during purification and TSA measurements. For fluorescence microscopy, pibrentasvir was added 6 hours after transfection of Huh7 cells by replacing the growth medium with fresh medium supplemented with 10 nM pibrentasvir.

#### SEC-MALS

FL WT protein was concentrated as described above and separated from minor impurities on a Superdex 200 10/300 column on an Äkta purification system (GE Healthcare) at 4°C. The column was preequilibrated with 100 mM tris-HCl (pH 7.0), 150 mM NaCl, 3 mM β-mercaptoethanol, 1% glycerol, and 0.05% DDM. Two peak fractions were collected and reconcentrated. SEC-MALS was performed at 4°C as described earlier (*36*) for 360 μg of protein at a flow rate of 0.5 ml/min. Concentration was determined at 20.5°C and 658 nm using a refractive index increment dn/dc of 0.143 ml/mg for DDM and 0.187 ml/g for NS5A-V5 as calculated by SEDFIT v16.1c (*37*).

#### CD spectroscopy

Circular dichroism (CD) was performed on a Jasco J-810 spectropolarimeter (Jasco GmbH) using 0.1-cm optical quartz cuvettes. Far-ultraviolet (190 to 260 nm) CD spectra of 0.5 μM NS5A FL and Δ2-3, 0.2 μM ΔAH and ΔAH-Δ2-3 constructs, and 22.6 μM AH peptide were measured at 20°C (at least three accumulations averaged). Proteins were purified in 25 mM sodium phosphate buffer (NaPi) (pH 7.5), 120 mM NaCl, 5% glycerol, and 2 mM β-mercaptoethanol plus 0.05% DDM for FL and diluted for measurements in 10 mM NaPi (pH 7.5) and 25 mM NaF ± 0.01% DDM. Curves were analyzed using GraphPad Prism 10 and smoothed using the smoothing function.

#### Thermal shift assay

To assess folding and stability of FL WT, truncations and mutants of NS5A ± lipids, TSA was performed where protein denaturation is indicated by increased fluorescence. Proteins at a final concentration of 250 ng/μl in buffer E (+0.05% DDM) were mixed with GloMelt (final concentration, 0.5×). Thermal unfolding was monitored using a StepOnePlus real-time polymerase chain reaction (PCR) machine (Applied Biosystems), gradual heating from 25° to 75°C in 0.1°C steps of 30 s each. For protein relipidation, liposome samples were prepared as for LRA with PC (100 mol %) or PC/PI4P (95/5 mol %) and used after dialysis. Ten nanomoles of liposomes dissolved in DDM [final detergent-to-lipid ratio (DLR) 6:1 (mol/mol)] was added per sample. All measurements were performed in triplicate. The melting curves shown are based on the subtraction of the values of control samples containing buffers with equivalent amounts of detergent ± native lipids.

#### Glycerol gradient

Equal amounts of 30 and 10% glycerol in phosphate-buffered saline (PBS) buffer were layered in ultraclear tubes and PC/Atto-PE/PI4P as for LRA ± 65 μg FL WT in PBS (PL ratio, 1:20). Samples were centrifuged in a SW60Ti rotor at 200,000*g* and 4°C for 20 hours. Fractions were collected top-to-bottom, and Atto550 fluorescence was measured in an EnVision reader (PerkinElmer) after solubilization with an equal volume of 20% DDM and incubation for 15 min at room temperature (RT).

#### Cross-linking

FL WT protein was purified without reducing agents. Before use, FL WT protein was centrifuged for 30 min at 186,000*g*. Two hundred fifty nanograms of protein was added per microliter reaction and incubated for 1 hour at RT. Cross-linking was initiated by adding freshly prepared BMH (1,6-bismaleimidohexane; Thermo Fisher Scientific) dissolved in DMSO. After incubation for 1 hour at RT, the reaction was stopped with 100 mM dithiothreitol for 15 min, and the cross-linking efficiency was analyzed using NuPAGE 3 to 8% midi gels (Thermo Fisher Scientific).

### Lipid binding assays

#### Protein-lipid overlay assay

PIP strips (Echelon Biosciences) were blocked with PBS containing 0.1% Tween and 3% of fatty acid–free bovine serum albumin (BSA; Roth) and incubated for 1 hour with (i) 11.82 nM NS5A FL or (ii) 5× molar amount (59.1 nM) of ΔAH, Δ2-3, or ΔAH-Δ2-3 in 5 ml of blocking solution. For detection of lipid-bound proteins/peptides, the membranes were incubated with mouse monoclonal anti–Strep-tag (IBA Lifescience; 1:10,000) and goat-anti-mouse horseradish peroxidase (HRP)–conjugated secondary antibodies (Bio-Rad; 1:5000), followed by addition of Immobilon Forte Western HRP Substrate (Millipore) and detection of chemiluminescence using Image Reader LAS 4000.

#### Liposome recruitment assay

For liposome preparation, PC/Atto550-PE (99.5/0.5 mol %) or PC/Atto550-PE/PA, PC/Atto550-PE/PI3P, PC/Atto550-PE/PI4P, PC/Atto550-PE/PI5P, and PC/Atto550-PE/PI(4,5)P_2_ (94.5/0.5/5 mol % each) dissolved in chloroform were mixed and dried for 5 min under N_2_ gas stream and 2 hours at high vacuum (20 mbar) at RT. Lipid films were dissolved in buffer D (buffer W supplemented with 3 mM β-mercaptoethanol) containing 1% octyl glucoside (OG) DLR of 8:1 (mol/mol). Liposomes were formed by rapid dilution of the lipid-detergent mixture with buffer D. OG was removed by dialysis 2 hours–overnight–2 hours against buffer D in 2 ml Slide-A-Lyzer MINI Dialysis devices 10 kDa MWCO (Thermo Fisher Scientific). Ready liposomes were mixed with ΔAH [protein-to-lipid ratio (PLR) 1:2000] in 1 ml of buffer D and incubated for 1 hour rotating at RT. Each sample was mixed with 2 ml of 60% Nycodenz (PROGEN) in buffer D, and step gradient was performed as in Malsam *et al.* (*38*). Liposomes with recruited protein were collected from the top of the gradients. After methanol-chloroform extraction, lipids were discarded, and proteins were dried, dissolved in 2× Laemmli buffer, separated by 10% SDS-PAGE with standard dilution of ΔAH, transferred to nitrocellulose membrane, detected with anti-Strep HRP-conjugated antibody (IBA Lifescience; 1:30,000), and quantified in Fiji/ImageJ (*35*). PIP strip assays were used qualitatively, whereas quantitative comparisons were obtained from liposome-based binding assays.

#### PI4P pull-down assay

NS5A ΔAH-Δ2-3 WT and K68E mutant protein were isolated from membrane fractions of infected Sf9 cells in the presence of DDM. Pull-down assay was performed according to the manufacturer’s instructions (Echelon Biosciences) with the following modifications: 7.5 μl of PI4P beads (P-B004A) was mixed with 1 μg of protein in a reaction volume of 20 μl (buffer E + 0.05% DDM) and incubated for 3 hours at RT under rotation. Beads were washed three times with 75 μl of buffer E + 0.05% DDM and heated to 70°C for 10 min upon addition of 10 μl of 2× LB. Samples were separated by 10% SDS-PAGE with standard dilution of ΔAH-Δ2-3, transferred to nitrocellulose membrane, detected with anti-Strep HRP-conjugated antibody (IBA Lifescience; 1:30,000), and quantified in Fiji/ImageJ (*35*).

### Liposome remodeling assay

Lipid films [PC/PE/Atto550-PE (67/32.5/0.5 mol %), PC/PE/Atto550-PE/PI4P (65/32.5/0.5/2 mol %), and PC/PE/Atto550-PE/PI4P/Chol (45/32.5/0.5/2/20 mol %)] were prepared as above, dissolved in buffer D containing 1% Triton X-100, and mixed with 300 μl of buffer E (+0.05% DDM) [DLR 4:1 (mol/mol)] (control) or FL WT or mutant protein containing AH in the same buffer [PLR 1:125 (mol/mol)]. To remove detergents, all lipid mixtures were incubated four times with 50 to 60 mg of wet Bio-Beads (soaked in buffer D) for 1 hour at RT. Bio-Beads were pelleted by centrifugation, and the supernatants were transferred to new tubes with fresh portions of Bio-Beads. All liposomes were purified by Nycodenz gradient as described above. Liposomes collected from the gradient were dialyzed for 2 hours–overnight–2 hours to remove the Nycodenz. Aliquots of control liposomes were incubated with ΔAH [PLR 1:250 (mol/mol)] or ΔAH-Δ2-3 [PLR 1:125 (mol/mol)] 1 hour rotating at RT. Liposomes obtained were analyzed by negative-stain transmission electron microscopy and cryo–electron microscopy/tomography (cryo-EM/cryo-ET).

#### Reconstitution of NS5A-AH

Lipid mixtures as for membrane remodeling and PC/PE/Atto550-PE/PA, PC/PE/Atto550-PE/PI3P, and PC/PE/Atto550-PE/PI(4,5)P_2_ (65/32.5/0.5/2 mol % each) were dried and dissolved as described above. Three hundred microliters of buffer E (+0.05% DDM) ± AH peptide was added per lipid mixture [PLR 1:300 (mol/mol)]. After detergent removal with Bio-Beads (see above), 15 μl of aliquots was analyzed by fluorescence microscopy (see below). The remainder of the samples was incubated overnight and applied to a Nycodenz gradient (see above). Proteoliposomes floating on the gradient were collected and 15 μl of aliquots analyzed by fluorescence microscopy (see below). The remainder of the liposomes was used for methanol-chloroform extraction of bound peptides. After isolation of AH peptides, they were separated by 16% SDS-PAGE, stained with Coomassie, and quantified using AH peptide standards in Fiji/ImageJ (*35*).

#### Negative-stain transmission electron microscopy

Carbon-coated copper grids were glow-discharged (15 mA for 45 s in a PELCO easiGlow system). Three microliters of sample was applied to the grid for 60 s and subjected to 3 cycles of side blotting and washing with 3 μl of buffer W. Negative staining was performed by applying 3 μl of 2% uranyl formate solution 3× for 15 s each. Samples were imaged at a nominal magnification of ×21,000 using a Tecnai Spirit BioTwin at 120 kV (Thermo Fisher Scientific). Electron micrographs were taken on a 14 μm/pixel US4000 charge-coupled device (CCD) camera (Gatan) with a final pixel size of 0.47 nm at the sample level.

#### Cryo-EM/Cryo-ET

Three microliters of sample was applied to a QF Cu/C, R2/2, 300 or 400 mesh copper grid (Quantifoil) or CF 2/1-3C Cu/C 300 mesh (Jena Bioscience). For cryo-ET, samples were mixed with 10-nm colloidal gold fiducials and applied to a 400-mesh QF R 2/2 gold grid with gold support (Quantifoil). The grids were glow-discharged twice (15 mA for 90 s in a PELCO easiGlow system) and cryoprepared using a Vitrobot Mark IV (Thermo Fisher Scientific). The grids were blotted with Whatman 595 filter paper, blotting force −2, at 8°C, and 100% humidity for a 5- to 11-s time range and immersed in liquid ethane. Two-dimensional imaging was performed on a 200-kV Glacios cryo–transmission electron microscopy (Thermo Fisher Scientific) with a Falcon III camera (Thermo Fisher Scientific) using the Thermo Fisher Scientific EPU software. Hole images were acquired with a defocus value of −50 μm at a magnification of ×13,500 with a pixel size of 1.06 nm. Recording magnification images were acquired with a defocus of −5 μm at a magnification of ×92,000 with a pixel size of 0.16 nm. The total electron dose used was 40 e−/Å^2^. For cryo-ET, tilt series were collected using a Titan Krios (Thermo Fisher Scientific) equipped with a K3 Summit direct electron detector (Gatan) with a Quantum energy filter (Gatan) operating at 300 kV. Uniaxial tilt series (−48° to +48°) were acquired using a hybrid dose-symmetric tilt scheme (*39*) with 3° intervals implemented in SerialEM (*40*). The electron dose for the untilted image (i.e., 0°) was increased (*41*) to 36 e−/Å^2^, while the remaining projections received a dose of 3.6 e−/Å^2^, acquired in 5 to 10 frames. The total exposure was ~150 e−/Å^2^. Images were taken at ×64,000 magnification, resulting in a pixel size of 1.4 Å/pixel. The nominal defocus was set between −2 and −5 μm in 0.25-μm steps. For tomogram reconstruction, per-tilt motion correction was performed using MotionCor2 (*42*), and defocus estimation was performed using Gctf for each projection (*43*). Tomographic tilt series were aligned using the 10-nm gold fiducials in IMOD (*44*). Tomographic reconstructions were generated using the weighted back projection implemented in IMOD (*44*). Images were used to measure liposome diameters in Fiji/ImageJ (*35*). The average of two longest perpendicular diameters was used for oval liposomes.

### Infectious HCV cell culture

Gaussia luciferase (GLuc) analysis of HCV replication was performed using the Renilla luciferase assay system (Promega) as previously described (*45*). H77S.3/GLuc and HCV-N.2/GLuc reporter viruses were prepared as previously described (*45*). To characterize the effect of NS5A mutations on RNA replication, targeted single and double mutations were made in derivatives of pN.2 (fig. S5, A to C, and table S2) (*46*). The seamless In-Fusion HD cloning method (Takara) was used to generate N.2/GLuc viruses carrying NS5A mutants, 68E and 66Q/68Q, using AflII- and BglII-digested vector and PCR-generated DNA fragments. In vitro transcription of HCV RNA was performed using the T7 RiboMAX Express Large Scale RNA Production System (Promega) as per the manufacturer’s protocol. Transfection of viral RNA was performed using the TransIT-mRNA Transfection Kit (Mirus) as described (*46*).

#### RNA interference

siRNA pools targeting PIP5K1B, PIP5K1C, and PI4KA and control siRNA pools were purchased from Dharmacon and Sigma-Aldrich (table S3). siRNA (20 nM) was transfected into cells using siLentfect Lipid Reagent (Bio-Rad) according to the manufacturer’s protocol.

### Immunocytochemistry and microscopy

Huh7 cells were cultured in Dulbecco’s modified Eagle’s medium (Gibco) supplemented with 10% fetal calf serum (Sigma Lifesciences) and penicillin/streptomycin (Sigma-Aldrich) at 37°C and 5% CO_2_. One day before transfection, 2 × 10^5^ cells per well were plated on a coverslip in a 12-well plate. Transfection of pcDNA3.1+ plasmids expressing FL WT or mutants with FuGENE HD transfection reagent (Promega) was performed according to the manufacturer’s protocol. The growth medium was replaced with fresh medium at 6 and 46 hours posttransfection. At 48 hours, cells were fixed with 4% paraformaldehyde for 20 min, permeabilized with 0.5% saponin for 3× 10 min, and blocked with 5% BSA. All reagents were dissolved in PBS. Anti-HCV-NS5A mouse antibody (ViroGen) was diluted 1:250 and incubated with the cells for 1 hour. Secondary anti-mouse Alexa555-conjugated antibody (Invitrogen; 1:500) was mixed with LDs stain LipidSpot 488 (Biotium; 1:1000) and Hoechst (final concentration, 2 μg/ml) and incubated with cells for 30 min. Cells were mounted with Dako fluorescent mounting medium. Images were captured the same day using a Zeiss Axiovert 100 inverted microscope equipped with a 100× lens and CCD camera. Images were processed and analyzed with Fiji/ImageJ software using the Common Tools Plugin from the Bioimaging and Optics Platform (BIOP) at the École polytechnique fédérale de Lausanne (EPFL) (*35*). The same settings were applied to all images in an experiment. Statistical analyses were performed with Prism V.9.5.0 (GraphPad, San Diego, CA). Data were expressed as median, 25th and 75th percentiles. Comparisons between groups were made using the nonparametric Mann-Whitney *U* test. Liposomes containing 0.5 mol % Atto550-DOPE with reconstituted AH peptide, prepared as described above, were mounted between a microscope slide and a coverslip, sealed, and imaged by fluorescence microscopy.

### Statistical analysis

Statistical significance was assessed by two-tailed *t* test, Mann-Whitney *U* test, Kruskal-Wallis test, or analysis of variance (ANOVA) as indicated in the figure legends, with *P* < 0.05 considered significant. Calculations were done using Prism 10 for macOS version 10.2.3 (GraphPad).
