# Supplementary material for: Phospholipid-driven conformational switching of HCV NS5A links protein folding to replication membrane remodeling
Source: Sci Adv. 2026 Apr 3;12(14):eaeb8863. doi: 10.1126/sciadv.aeb8863 (PMC13048264; doi:10.1126/sciadv.aeb8863)
Supplement: Supplementary file 1 — Figs. S1 to S11 Tables S1 to S3 Legends for movies S1 and S2 [file sciadv.aeb8863_sm.pdf]

Science Advances
AAAS

# Supplementary Materials for

## Phospholipid-driven conformational switching of HCV NS5A links protein folding to replication membrane remodeling

Anna V. Bulankina *et al.*

Corresponding author: Christoph Welsch, welsch@med.uni-frankfurt.de

*Sci. Adv.* **12**, eaeb8863 (2026)
DOI: 10.1126/sciadv.aeb8863

**The PDF file includes:**

Figs. S1 to S11
Tables S1 to S3
Legends for movies S1 and S2

**Other Supplementary Material for this manuscript includes the following:**

Movies S1 and S2

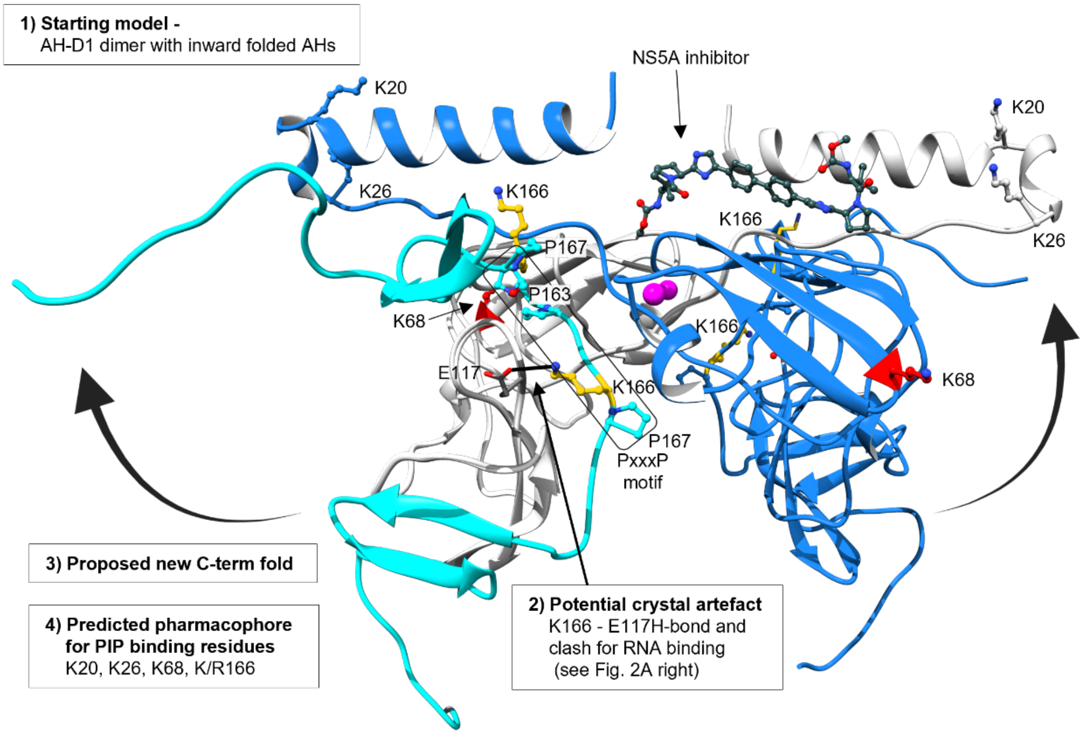


**Figure S1. Starting model of the NS5A AH-D1 dimer, conformational rearrangements and PIP-binding pharmacophore prediction.** The starting model is based on the AH–D1 dimer structure published by Nettles *et al.* (*11*). Existing crystal structures constrain the D1 C-terminal region through an internal E117–K166 hydrogen bond. Using torsional flexibility within a PxxxP linker (residues 160–170), we proposed alternative C-terminal orientations. These rearrangements reveal a putative phospholipid-binding pharmacophore in D1 and the AH.

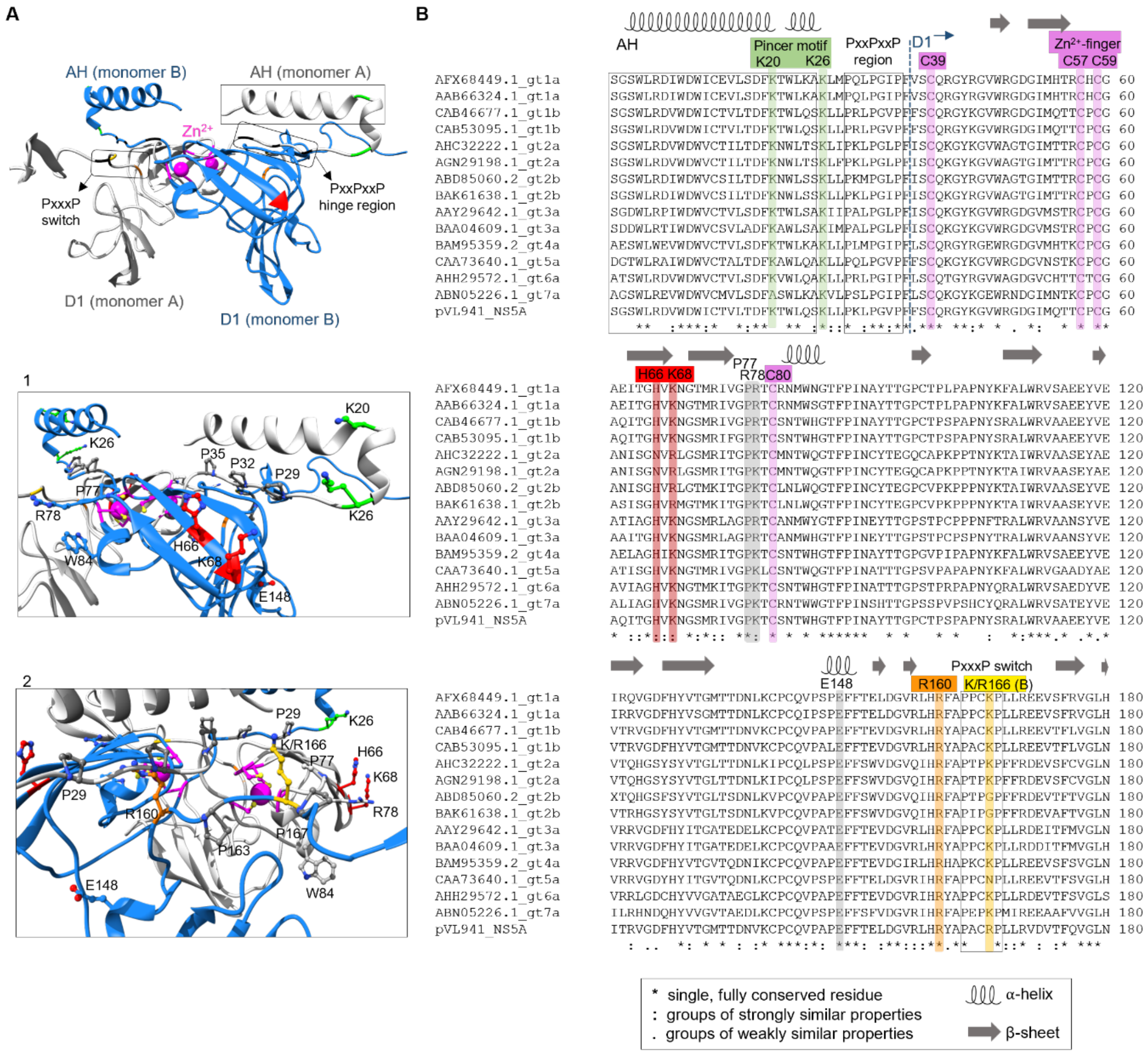

**Figure S2. Structural informatics and polyproline hinge regions. (A)** Structure of NS5A dimer model with new C-term fold and detail views. Predicted phospholipid-interacting residues for mutagenesis are highlighted, K68 (red) and shown in detail views, R160 (orange) and K/R166 (yellow). **(B)** Sequence alignment of AH-D1 from different HCV genotypes/subtypes computed with Clustal Omega (https://www.ebi.ac.uk/Tools/msa/clustalo/) (*48*); N.2 (gt1b) is used in this study (pVL941_NS5A); $Zn^{2+}$ binding residues (magenta), pincer residues K20-K26 (*17*) (green), other residues (gray). Secondary structure elements are indicated by helices (for α-helices) and arrows (for β-strand/-sheets).

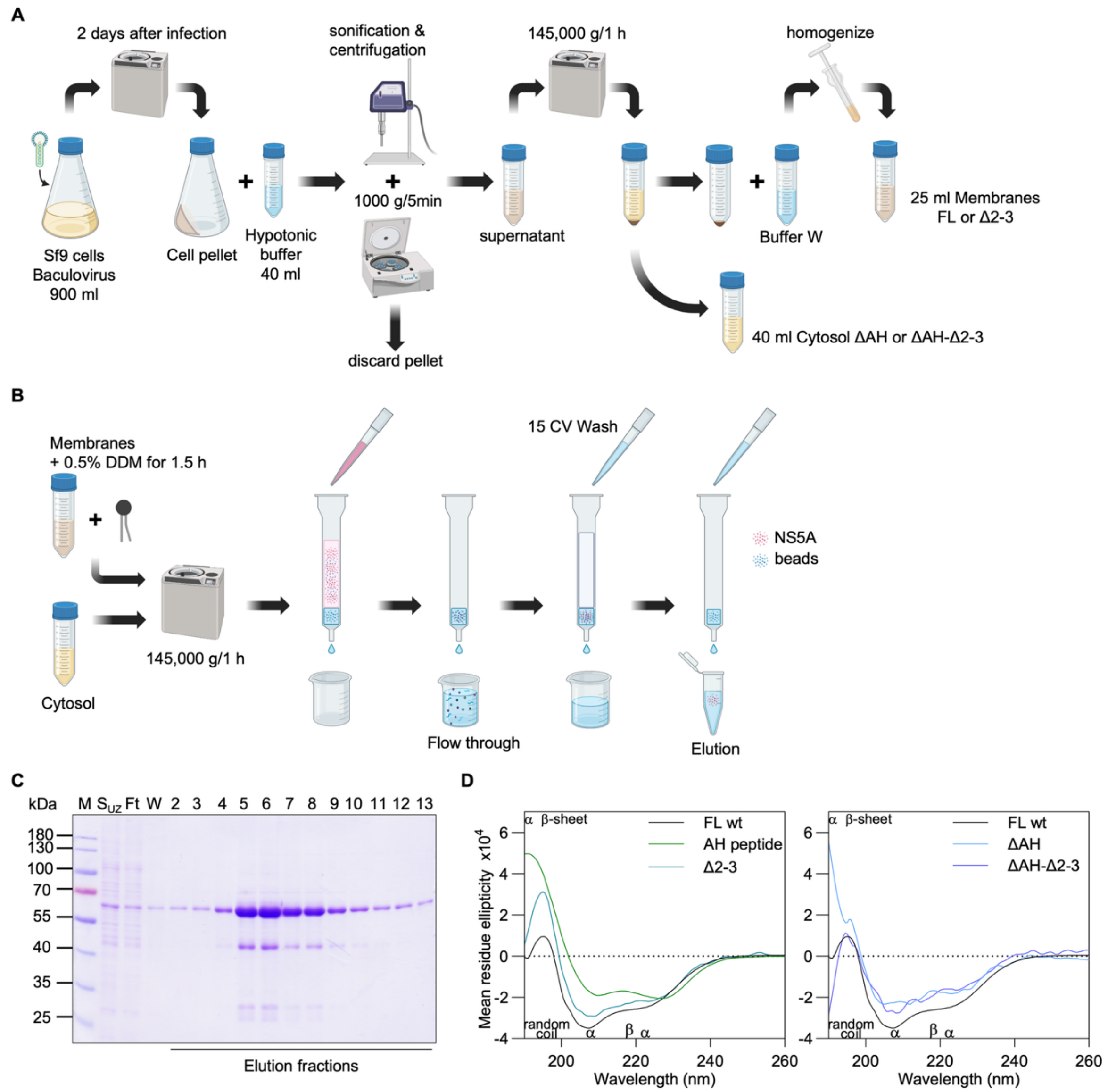


**Figure S3. Expression and purification of NS5A constructs. (A)** Expression in Sf9 cells using P2 baculovirus with subsequent membrane and cytosol preparation, respectively. Created in BioRender. Welsch, C. (2025) https://BioRender.com/3vk1az7. **(B)** Solubilization and purification of NS5A protein using Strep-tag affinity chromatography. Created in BioRender. Welsch, C. (2025) https://BioRender.com/7gc2zpr. **(C)** Representative Coomassie stained SDS-PAGE of FL wt protein expressed in Sf9 cells, purified by Strep-tag affinity chromatography. $S_{UZ}$ – Supernatant lysate loaded on affinity chromatography, Ft – flow through, W – wash step, 2-13 – elution fractions. The purity of the protein produced is 85-95%. **(D)** Circular dichroism (CD) spectra of purified FL wt and protein constructs. AH peptide: α-helical structures (*17*); FL wt: mixture of α-helices, β-sheets, and disordered regions, comparable to the spectra previously shown by Kwon *et al.* (*18*). Deletion of AH (ΔAH and ΔAH-Δ2-3, right) results in less pronounced α-helical features, as described by Gupta *et al.* (*49*), whereas deletion of disordered domains 2 and 3 (Δ2-3 and ΔAH-Δ2-3) results in more pronounced β-sheets and comparable expression of the α-helical features.

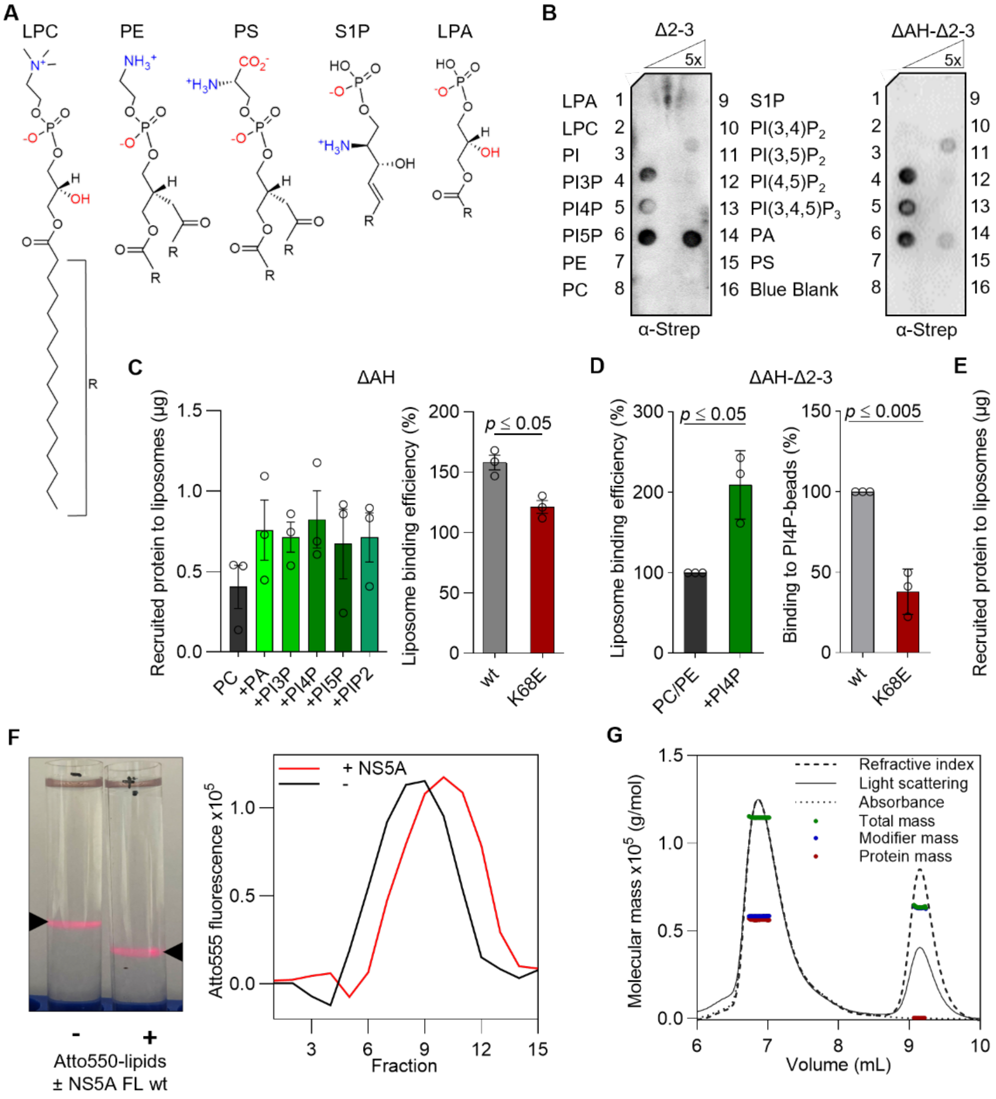

**Figure S4. Biochemical characterization of NS5A protein isolated from insect cells. (A)** Lipid molecules from PIP strips not shown in Figure 2: PE, phosphatidylethanolamine; PS, phosphatidylserine, S1P, sphingosine-1-phosphate; LPA, lysophosphatidic acid; LPC, lysophosphocholine. **(B)** PLOAs with phospholipid-binding patterns for Δ2-3 and ΔAH-Δ2-3 (each 5-fold molar amount). Anti-Strep antibody was used to immunodetect NS5A. **(C)** Quantification of SDS-PAGE of ΔAH wt recruitment to different lipid compositions (left; see also Fig. 2H) and LRA of wt and K68E mutant to PI4P containing liposomes. **(D)** LRA of ΔAH-Δ2-3 wt to PC/PE control and PI4P containing liposomes (left) and quantified binding of ΔAH-Δ2-3 wt and K68E mutant to PI4P-beads (see Fig. 2F). **(E)** Quantification of SDS-PAGE of AH peptide reconstituted into different lipid compositions (see also Fig. 2G). **(F)** Reconstitution assay of FL wt with PC/Atto-PE/PI4P liposomes separated by liposome flotation in a glycerol density gradient (left) and Atto550 fluorescence after fractioning of the gradient ± NS5A (right). **(G)** SEC-MALS of FL wt isolated from Sf9 cells in the presence of detergent DDM. Peak 1: NS5A monomer plus DDM micelle with 114,600 Da; the molecular weight of NS5A FL is 56,250 Da. Peak 2: DDM micelle with a molecular weight of 58,350 Da. Data shown are mean ± s.e.m. and unpaired two-tailed *t*-test for C and D. One-way Anova was used for E.

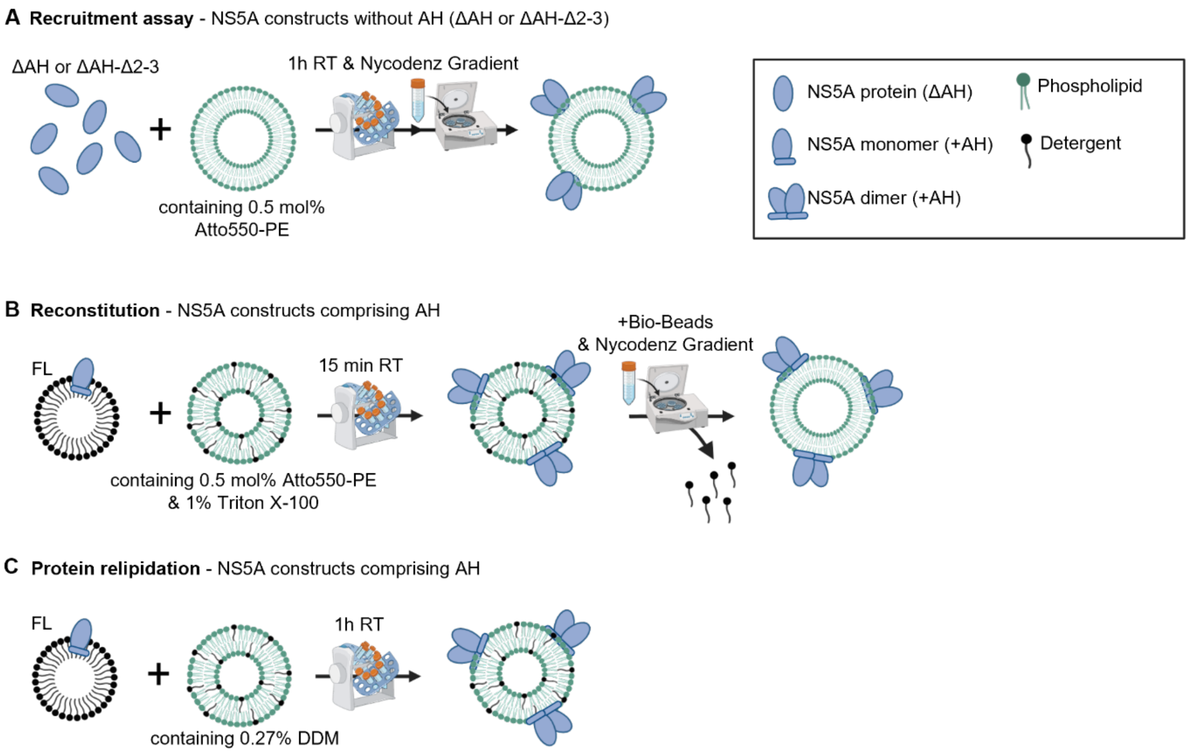


**Figure S5. Schematic of liposome and protein relipidation assays. (A)** In the liposome recruitment (LRA), liposomes are prepared and then mixed with NS5A protein lacking the AH domain, which is isolated without detergent. **(B)** In the liposome reconstitution and remodeling assays, NS5A containing the AH domain is isolated in the presence of detergent, while phospholipids are solubilized in a detergent-containing buffer to form mixed micelles. After combining the protein and native lipids, detergent is removed using Bio-Beads, resulting in the formation of proteoliposomes containing reconstituted proteins. **(C)** In the protein relipidation assay, NS5A protein isolated in detergent-containing buffer is mixed with detergent-solubilized lipids to form mixed micelles. Created in BioRender. Welsch, C. (2025) https://BioRender.com/78gfz0v.

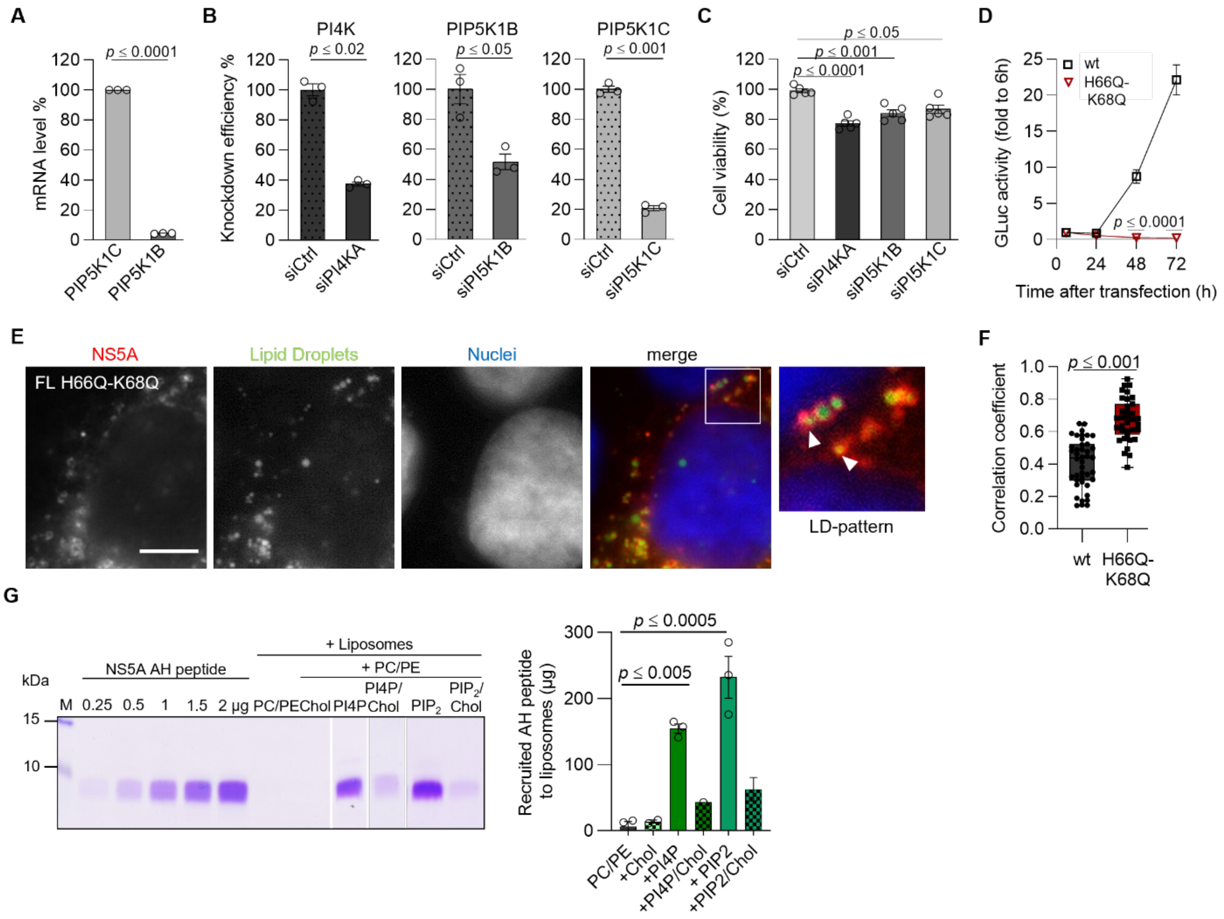


**Figure S6. Impact of PI4P metabolism, redistribution of the NS5A mutant H66Q-K68Q and impact of cholesterol on NS5A AH-phospholipid interaction.** **(A)** Relative mRNA abundance of *PIP5K1B* and *PIP5K1C* in Huh7.5 cells. **(B)** Efficiency of siRNA KD for indicated enzymes. **(C)** Cell viability in siRNA-transfected cells as measured by WST8 assay. **(D)** Replication capacity of the mutant virus H66Q-K68Q in N.2 (gt1b) genetic background. **(E)** Immunofluorescence images showing the subcellular distribution of NS5A 48 hours after transfection of Huh7 cells with NS5A H66Q-K68Q mutant (LD pattern); NS5A (red), LDs (green) and cell nuclei (blue). Scale bar is 5 µm. **(F)** Pearson's correlation for the association of NS5A with LDs shown in (E). **(G)** NS5A-AH standard and peptide recruited to and extracted from liposomes with different lipid composition (cholesterol, PI4P- or PI(4,5)$P_2$-containing liposomes). Quantification of SDS-PAGE on the right. Data shown as mean ± s.e.m. for A-D, F; quartiles (dashed lines) and median (solid lines) for F. $n$ = 3 or 5 biologically independent samples (wells/gels). Unpaired two-tailed $t$-test for A and B, one-way ANOVA for C, and two-way ANOVA for F.

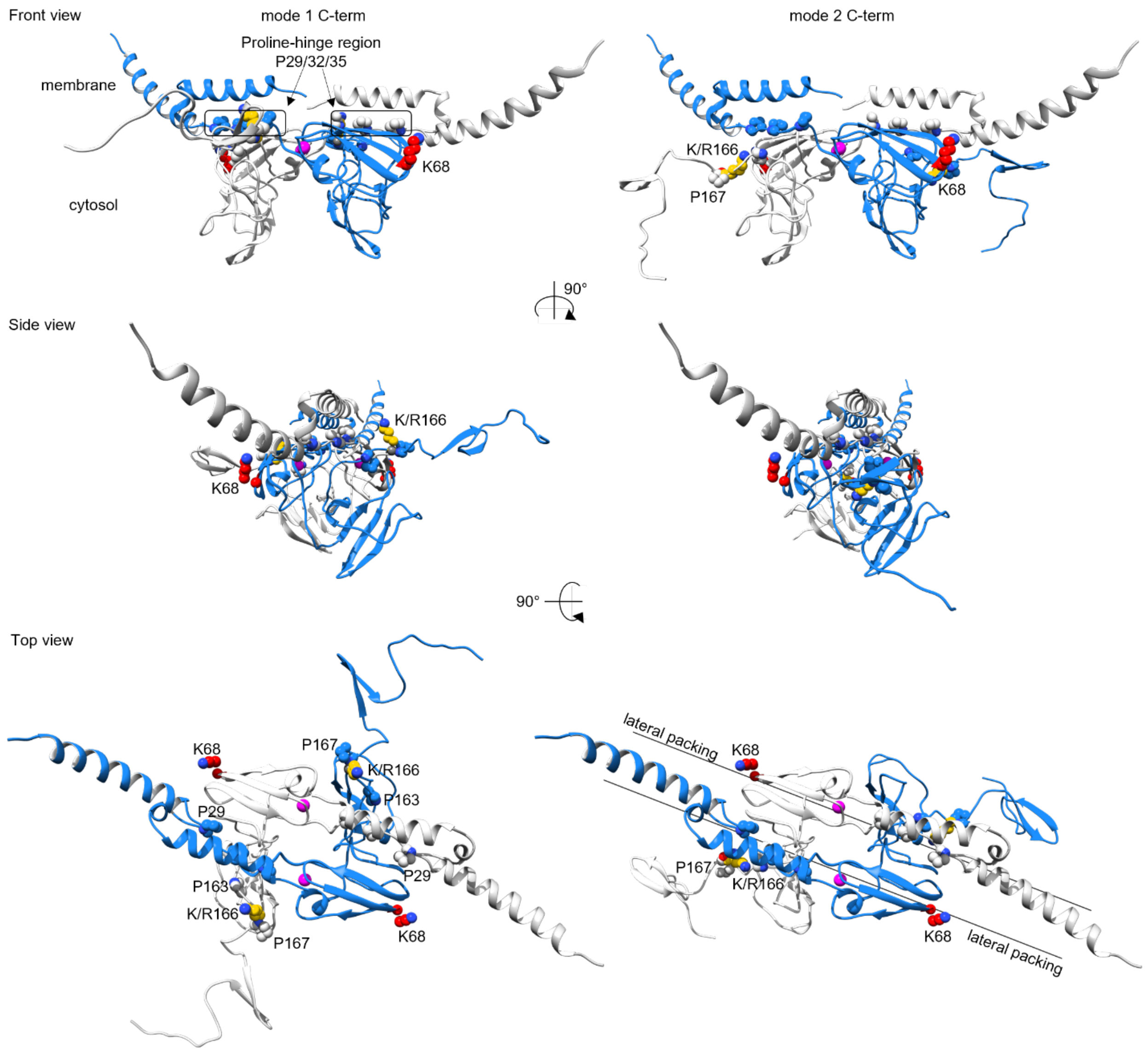


**Figure S7. Mechanistic model of NS5A fold transitions and lateral packing.** Different views of the C-terminal conformational rearrangements, front, side and top view; mode 1 (left) and mode 2 (right) of the C-term fold. Key residues are shown as CPK models. Top view showing the lateral packing (indicated as lines), stabilizing the membrane-proximal AH-D1 dimer interface.

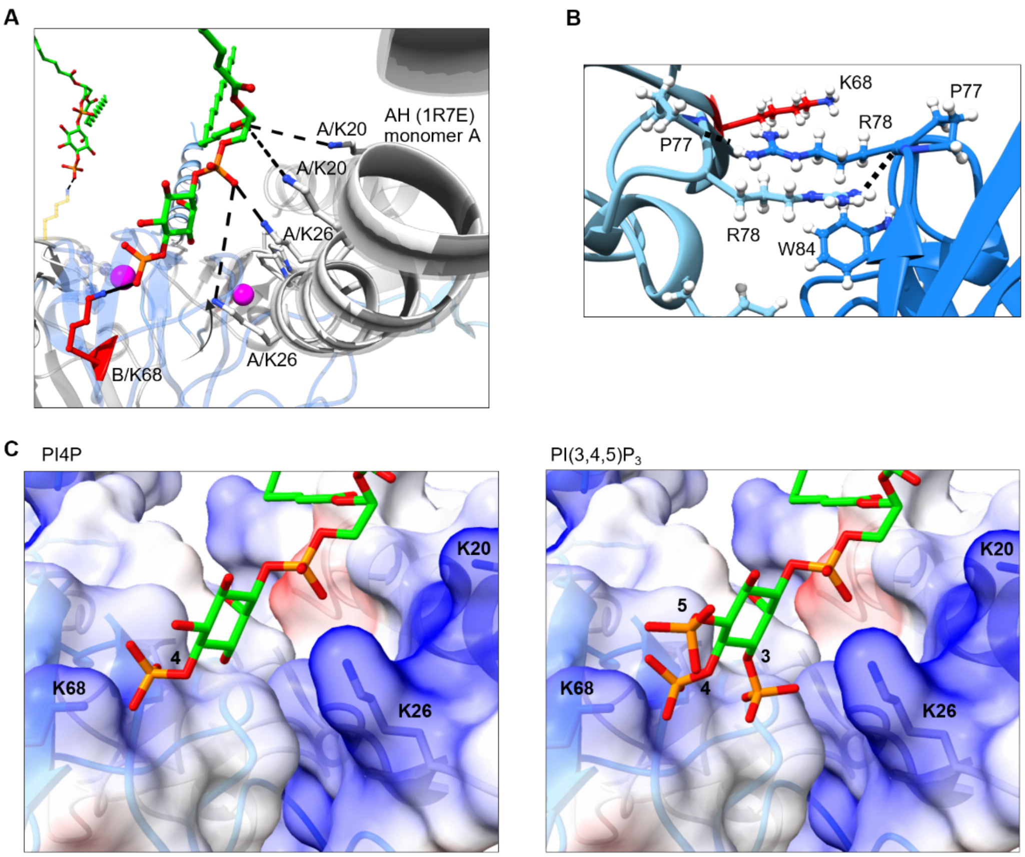


**Figure S8. Details of the dimer interface and PIP-binding site. (A)** Close up-view of a PIP-binding site highlighting the cooperative roles of K68 and the AH in enabling high-affinity binding (see also Fig. 5, middle inlet). **(B)** Close up-view of the NS5A dimer interface residues highlighting P77-R78 H-bond interactions (see also Fig. 4I). **(C)** Mono- and triphosphorylated PIP headgroups positioned into the pharmacophore pocket, revealing a highly selective electrostatic complementarity for the 4-phosphate with residue 68, whereas the 3- and 5-phosphates lacked corresponding interaction partners.

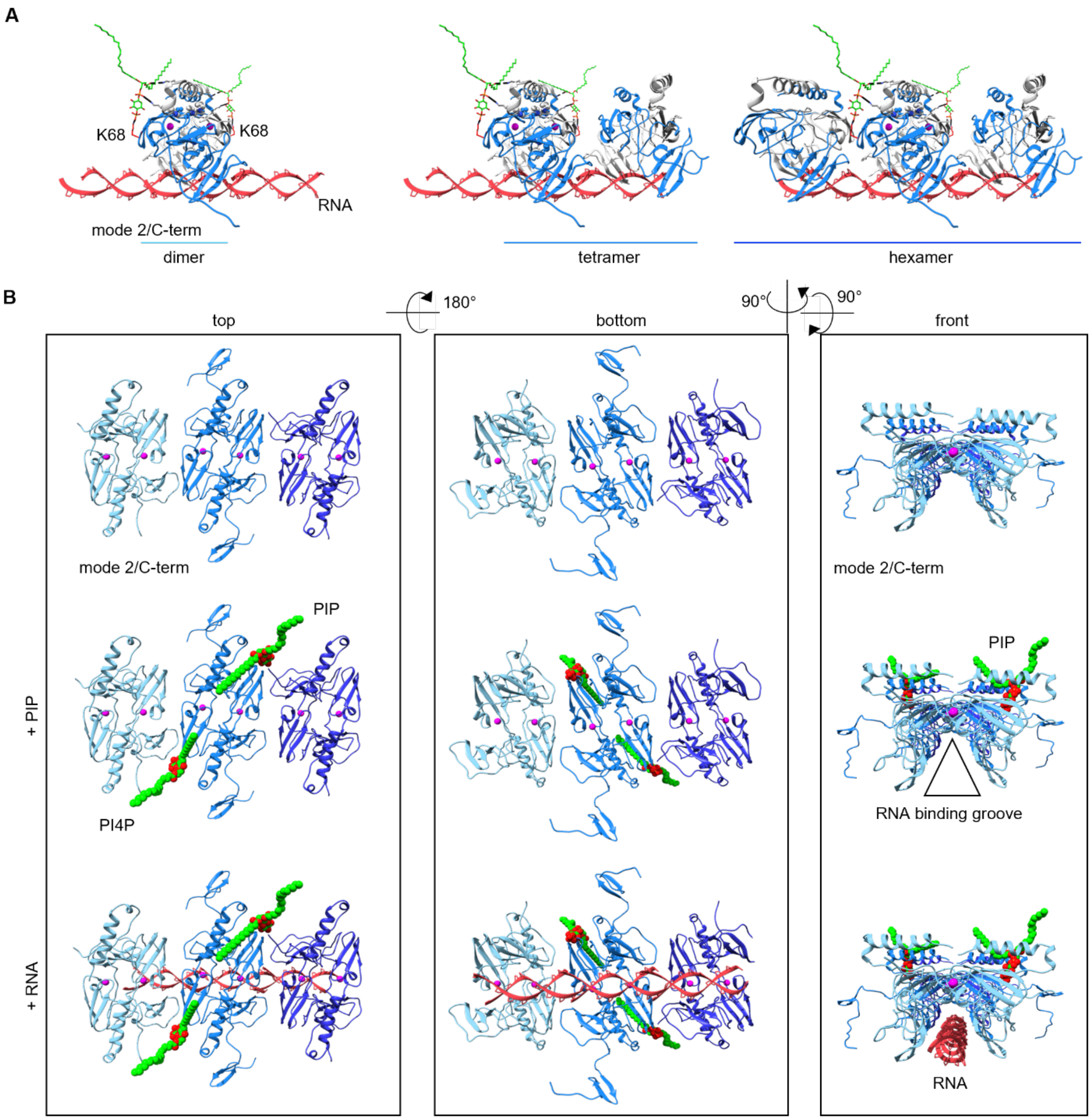


**Figure S9. NS5A hexamer and RNA-binding groove model. (A)** The new C-term folds (*mode 2*) of NS5A allow for further oligomerization aligned along RNA. **(B)** NS5A hexamer model from different angles with stepwise addition of PIPs (green) and RNA (red). $Zn^{2+}$ ions are shown in magenta. PI4P was used as model ligand.

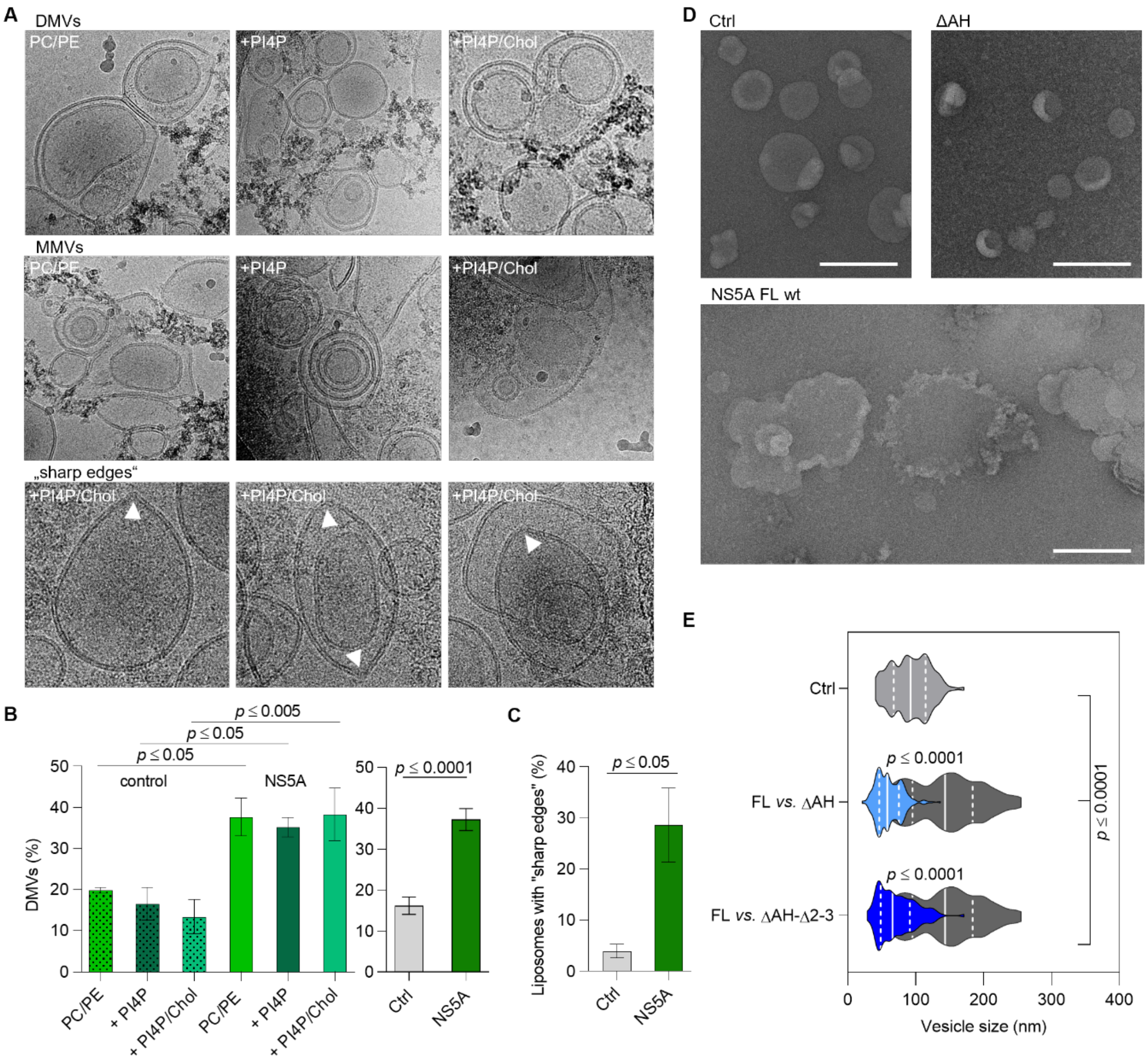


**Figure S10. Proteoliposome remodeling. (A)** Cryo-EM images of liposomes with different lipid composition; DMVs/MMVs (top, middle) and 'sharp edges' (bottom) induced by FL wt. **(B)** Quantification of DMVs in cryo-EM on liposomes ± reconstituted FL wt with different lipid composition: PC/PE, PC/PE/PI4P and PC/PE/PI4P/Chol (left). Comparison of DMV formation with and without NS5A protein (right). **(C)** Frequency of 'sharp edges' on liposomes containing PC/PE/PI4P/Chol ± FL wt. **(D)** Negative stain TEM images from control liposomes, ΔAH and FL wt. Scale bar is 100 nm. **(E)** Violin plot of curvature induction by ΔAH (light blue) and ΔAH-Δ2-3, blue in nsTEM experiments. Data shown are mean ± s.e.m. for B and C; quartiles (dashed lines) and median (solid lines) for E. In A-E, $n = 2$ biologically independent samples (grids). Unpaired two-tailed $t$-test for B and C. Kruskal-Wallis test for E.

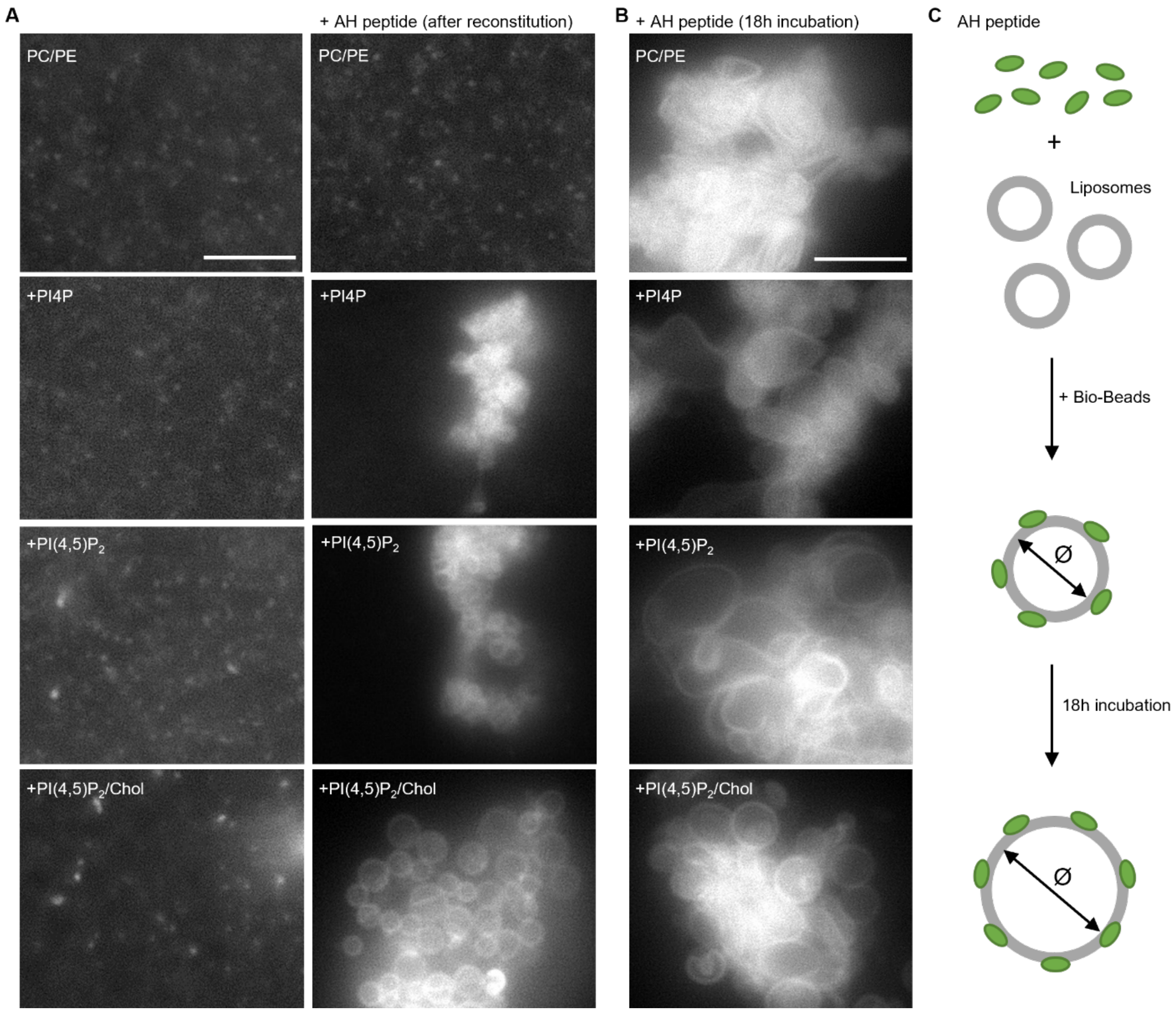


**Figure S11. Lipid recruitment by AH peptide.** Light microscopy time course analysis of PC/PE liposomes with 0.5% Atto550-labeled PE ± PIPs and cholesterol. **(A)** Liposomes immediately after reconstitution (removal of Bio-Beads) ± AH peptide. *n* = 3 biologically independent samples (wells). **(B)** Additional incubation of liposomes with AH peptide for 18 h. Scale bar 5 µm. *n* = 3 biologically independent samples (wells). **(C)** Schematic of lipid recruitment to liposomes by AH peptide. The observed increase in liposome size may reflect a combination of lipid recruitment and vesicle fusion events mediated by the AH peptide; these two processes cannot be unambiguously distinguished under the present experimental conditions.

## Tables

**Table S1: Forward primers used for cloning/mutagenesis.**

| Mutation/Construct | Sequence (5´ - 3´) |
|---|---|
| ΔAH | CGGGCCATGGGGCTTGCCGGGAGTCC |
| Δ2-3 | CCATGACTCCTAATAATAACCGGACGCTGACC |
| K20A-K26A | GACTGACTTCGCGACCTGGCTCCAGTCCGCGCTCCTGCCG |
| H66Q-K68Q | CACAAATTACCGGACAGGTCCAAAACGGTTCCATGAG |
| K68E | CCGGACATGTCGAAAACGGTTCCATGAG |
| R160E | GGGTGCGGTTGCACGAGTACGCTCCGGCGTG |
| R166E | CGCTCCGGCGTGCGAACCTCTCCTACGGG |

Modified codons are underlined.

**Table S2: Primers used for RT-qPCR (in HCVcc).**

| Gene | Forward | Reverse |
|---|---|---|
| PI4KA | GTCTGATGTGGCCTATAGGG | TCAGGCATGGGATAGCATAC |
| PIP5K1B | TCTCTGGTGCCTGATACCTC | GTAGTGATGGGTGGCCTACT |
| PIP5K1C | TCTTTCGAAGAAGCCACTAC | GTAATCTGCTGCAGATCCTC |

**Table S3: siRNAs to target phospholipid metabolism.**

**siRNAs from Dharmacon**

| Gene Symbol | Sequence |
|---|---|
| PIP5K1B | GCAGACAGAUUUCUUAAGU |
| PIP5K1B | UAAGACAUACGCUCCAUUA |
| PIP5K1B | CGACAGGCCUACACUCUAU |
| PIP5K1B | GGCCUACACUCUAUUCAAA |
| PIP5K1C | CCAAAUUCCUGUACUGUAA |
| PIP5K1C | GGCAAGACCUAUUUAUAAU |
| PIP5K1C | GGAGAUAUACUUGGUGUUG |
| PIP5K1C | GAUAGAAGUCUGUAAAUAC |

**siRNAs from Sigma**

| Gene Symbol | Sequence |
|---|---|
| PI4KA | CCCUAAAGGCGACGAGAGA |

**Supplementary movie 1.** Overlay of PDB 1ZH1 and the predicted AH-D1 structure showing movement of D1 C-terminus towards the membrane.

**Supplementary movie 2.** Folding of NS5A-AH and C-terminus of D1 during NS5A assembly.
